# Supply Chain Events and Risk of Drug Shortage in Canada

**DOI:** 10.1001/jamanetworkopen.2026.16632

**Published:** 2026-06-04

**Authors:** Araniy Santhireswaran, Martin K. H. Ho, Katherine Callaway Kim, Shanzeh Chaudhry, Katie J. Suda, Etienne Gaudette, Lisa Burry, Mina Tadrous

**Affiliations:** 1 Leslie Dan Faculty of Pharmacy, University of Toronto, Toronto, Ontario, Canada; 2 Division of General Internal Medicine, Department of Medicine, University of Pittsburgh School of Medicine, Pittsburgh, Pennsylvania; 3 Center for Healthcare Evaluation, Research, and Promotion, VA Pittsburgh Healthcare System, Pittsburgh, Pennsylvania; 4 Institute of Health Policy, Management and Evaluation, University of Toronto, Toronto, Ontario, Canada; 5 Sinai Health and Lunenfeld-Tanenbaum Research Institute, Toronto, Ontario, Canada; 6 Women’s College Hospital, Toronto, Ontario, Canada

## Abstract

**Importance:**

Drug shortages are an ongoing challenge in health care delivery. Research has focused on factors associated with manufacturer-level shortage reports; however, these reports do not reflect population-level drug availability. It is essential to determine the impact of supply chain events on trends in drug utilization and identify factors associated with decreases in use to ascertain shortage risk and inform future policy prioritization.

**Objective:**

To quantify incident shortages following Canadian supply chain events and determine drug characteristics associated with increased shortage risk.

**Design, Setting, and Participants:**

This matched cohort study analyzed monthly trends in drug purchasing of Canadian pharmaceutical products from the IQVIA MIDAS database from January 2017 to December 2021 with a 6-month follow-up. Data were analyzed from September 2023 to September 2024.

**Exposure:**

Reporting of a supply chain issue in Drug Shortages Canada.

**Main Outcomes and Measures:**

The main outcome was an incident shortage, defined as a decrease of 33% or more in purchase volume within 6 months following the supply chain issue. A logistic regression with random effects compared the odds of experiencing an incident shortage for cases and controls. A secondary outcome, shortage intensity, defined as the decrease in purchases multiplied by the duration, was analyzed using a zero-inflated beta regression with random effects.

**Results:**

Of 1591 drugs, 972 unique drugs (61%) were exposed to 1919 supply chain events. Incident shortages were observed in 11% of cases (216 drugs), compared with 7% of controls (1271 comparators). Drugs with baseline sales less than $100 000 (odds ratio [OR], 4.01; 95% CI, 3.03-5.30), anti-infective drugs (OR, 3.07; 95% CI, 2.33-4.04), and over-the-counter drugs (OR, 3.05; 95% CI, 2.38-3.90) had greater odds of experiencing an incident shortage. Schedule G drugs, which include controlled drugs (OR, 3.61; 95% CI, 1.66-7.77), drugs with more than 25 unique products available (OR, 2.86; 95% CI, 1.89-4.44), and those with equal dominance in both retail and hospital sectors (OR, 2.43; 95% CI, 1.93-3.01) had the largest magnitudes of association with higher shortage intensity.

**Conclusions and Relevance:**

In this study of shortage incidence following Canadian supply chain events, 9 in 10 reported supply chain events did not experience true shortages. These findings highlight a need for a unified shortage definition focusing on real disruptions in supply and can be used to guide policies to manage drug shortages, improve patient outcomes, and enhance health care delivery.

## Key Points

**Question:**

What is the incidence of drug shortages following Canadian supply chain events, and what drug characteristics are associated with an increased risk of a drug shortage?

**Findings:**

In this matched cohort study, 972 unique drug inventories were exposed to 1919 supply chain events, and 9 of 10 supply chain disruptions did not experience an incident shortage (≥33% decrease). Drugs with baseline sales less than $100 000, anti-infective drugs, and over-the-counter drugs had significantly greater odds of experiencing an incident shortage.

**Meaning:**

These findings suggest that a supply chain issue alone does not translate into a shortage in available drug supply, highlighting a need for a better system to identify true shortages that affect patients’ ability to access medications.

## Teaser

This cohort study quantifies incident drug shortages following supply chain events in Canada and examines drug characteristics associated with increased shortage risk.

## Introduction

Drug shortages continue to increase across the world; global drug shortages have increased by 101% since 2021.^1,2,3^ Health Canada defines a drug shortage as a situation where the drug manufacturer is unable to fulfill drug orders on time.^4^ Shortages can occur for various reasons, from manufacturing challenges, unexpected demand spikes, and economic and regulatory issues.^5,6^ Disruptions in drug supply force patients to alter or even discontinue treatment, leading to negative clinical outcomes.^7,8,9^ To minimize shortage impacts, many policy measures have been implemented.^4,10,11^ However, supply chain disruptions continue to hinder patient access to medicines.^12,13^

In March 2017, Health Canada mandated manufacturers to report all known upstream supply chain issues that could lead to downstream shortages.^14,15^ All drug shortages and discontinuations must be reported within 5 calendar days of the manufacturer becoming aware of supply chain issues.^14^ To understand how shortages manifest and the associated risk factors, many studies have analyzed drug shortage reports.^16,17,18^ However, reports at the manufacturer-supply level do not reflect downstream population-level drug supply and the consequent impact on patient-level access, use, and health outcomes. It is therefore important to understand the impact of supply chain events on available drug supply and population-wide access. Moreover, identifying which drugs are more likely to be in short supply is crucial. The aim of this study is to quantify the frequency of population-level drug shortages following Canadian supply issue events and determine drug characteristics associated with an increased risk of a shortage. This study addressed the question of whether reported supply issues in Canada are associated with measurable population-level shortages and which drug characteristics are associated with increased shortage risk.

## Methods

We conducted a matched cohort study using Canadian drug purchasing data from January 2017 to December 2021. The conceptual model used in this study was developed and validated in earlier work.^19^ The model emphasizes that a supply chain issue report is not a direct indicator of a shortage in the available drug supply at the population level. Thus, not all supply chain issue reports will lead to a significant drug shortage.^19^ This study did not involve human participants; therefore, in accordance with the Tri-Council Policy Statement,^20^ institutional ethics approval and informed consent were not required. This study follows Strengthening the Reporting of Observational Studies in Epidemiology (STROBE) reporting guideline for cohort studies.

### Data Sources and Study Population

Our drug cohort included pharmaceutical products purchased in Canada from 2017 to 2021 in the IQVIA MIDAS dataset. IQVIA MIDAS reports monthly drug sales and purchase data from health systems, retail pharmacies, and major retail outlets (mass merchandisers, grocery stores, and convenience stores without pharmacies) for prescription and over-the-counter (OTC) drugs at the national level (eTable 1 in Supplement 1). Drug purchase volumes are reported in standardized units, where 1 standardized unit represents the smallest purchased unit of a drug (ie, 1 tablet, capsule, 1 intravenous vial, or 5 mL oral liquid).

A unique drug was defined as a unique ingredient list and broad formulation category (eTable 2 in Supplement 1). We excluded drugs with incomplete purchasing data, radiopharmaceuticals, unapproved or special access drugs, allergens, diagnostic tests, natural products, and nonpharmaceutical preparations. All drugs meeting inclusion criteria during study period were included in analysis. This dataset includes data on the number of manufacturers and the total wholesale value of drug sales.^21^ IQVIA MIDAS monthly purchases data for Canada are used as a proxy to measure the total Canadian drug supply. IQVIA MIDAS data have been used for many other studies and have high accuracy.^22^ The annual internal validation audit conducted by IQVIA reported 99.7% to 100% precision for Canadian data.

We included supply chain reports issued from March 2017 (the start of mandated reporting) to September 2021 (3 months prior to the end of data) from the Drug Shortages Canada website.^23^ Reports of all statuses were included, and exposure time was the report creation date in calendar time. Reports for the same drug formulation with fewer than 90 days between the end date of one report and the start date of the next were merged and considered a single supply chain issue event.^19^ For discontinuation reports and shortage reports with missing end dates, the end date was imputed as 90 days after the report creation date. Drug information was obtained from other data sources including the Health Canada Drug Product Database, ICES drug list, World Health Organization (WHO) full electronic essential medicines list database and Health Canada Tier-3 historical list and website. All data sources and covariates are detailed in eTable 1 and eTable 2 in Supplement 1. For outcome data, drugs with no sales data were excluded by design, and all included drugs had complete covariate data with no missing values.

### Matching Approach

Exposure density sampling (EDS) was used to match each supply chain issue event with a random sample of 10 comparator drugs with no supply issue at the time of exposure.^24,25,26,27^ Previous studies using a similar study design have employed a 10:1 control to case ratio.^19^ Unlike incidence density sampling, used in nested case-control studies, EDS matches at time of exposure rather than time of outcome; this controls for seasonal fluctuations in drug purchases and when combined with multivariable confounder adjustment, leads to unbiased results.^19^ We did not match based on baseline purchasing trends or other drug characteristics because many supply chain disruptions are unexpected in nature, and matching on these factors would reduce the heterogeneity needed to evaluate drug characteristics associated with shortages. EDS allows drugs to appear more than once in each group, where the same drug can be exposed multiple times and can be a comparator during unexposed time periods.

### Outcomes

The primary outcome was an incident shortage, defined as at least a 33% decrease in purchases within 6 months following the supply chain issue compared with the 3 months prior (eFigure 1 in Supplement 1). This 33% threshold has been used in previous studies in the US and Canada,^19,28,29^ and was further validated in our data, showing 90.5% specificity (eTable 3 in Supplement 1). A supplementary analysis examined severe incident shortages, defined as at least a 66% decrease (eFigure 1 in Supplement 1). The secondary outcome was a continuous shortage intensity measure, defined as the reduction in drug purchases multiplied by the duration of the decrease in months (eFigure 2 in Supplement 1).^30^ Differences in outcomes by drug characteristics were assessed conditional on supply chain issue exposure status.

### Statistical Analyses

#### Primary Analysis

A random-effects logistic regression compared incident shortage odds for drugs with and without a supply chain issue. A zero-inflated beta regression with random effects compared odds of a shortage intensity of 0 vs nonzero, and odds of higher intensities. This model was chosen for the continuous outcome and accounts for the large proportion of zero values in our data.^31^ Covariates were included for anatomical therapeutic chemical class (ATC), formulation, unit price, number of manufacturers, baseline sales, WHO essential medicine status,^32^ therapeutic equivalents, time on market, active pharmaceutical ingredients, Tier 3 status,^4^ drug identification numbers (DINs), drug schedule, brand vs generic, previous supply chain issue, dominant sector (retail vs hospital) and Ontario drug benefit (ODB)^33^ coverage (eTable 2 in Supplement 1). Model fit was determined using Akaike information criterion corrected and bayesian information criterion by removing any potentially correlated variables.^34,35,36^ A random intercept was included for each matched set to account for dependency introduced by matching. Interactions were considered between unit price and formulation but were not included due to perfect separation. Manufacturer market share variable was considered but excluded given strong correlation with number of manufacturers. Analyses were conducted from September 2023 to September 2024 in SAS Studio 3.81 (SAS Institute) and R Studio 4.4.1 (Posit). Significance was assessed at a type I error rate of .05 with no adjustments for multiple testing.

#### Sensitivity Analyses

For the primary outcome, we excluded discontinuation reports, used 5 comparators instead of 10, assessed the outcome in the 9 months instead of 6 months, and included a COVID-19 flag post–March 2020. For the secondary outcome, we examined a categorical shortage intensity, with categories 0, greater than 0 to 0.1, greater than 0.1 to 0.4 and greater than 0.4.

## Results

**Figure 1.**
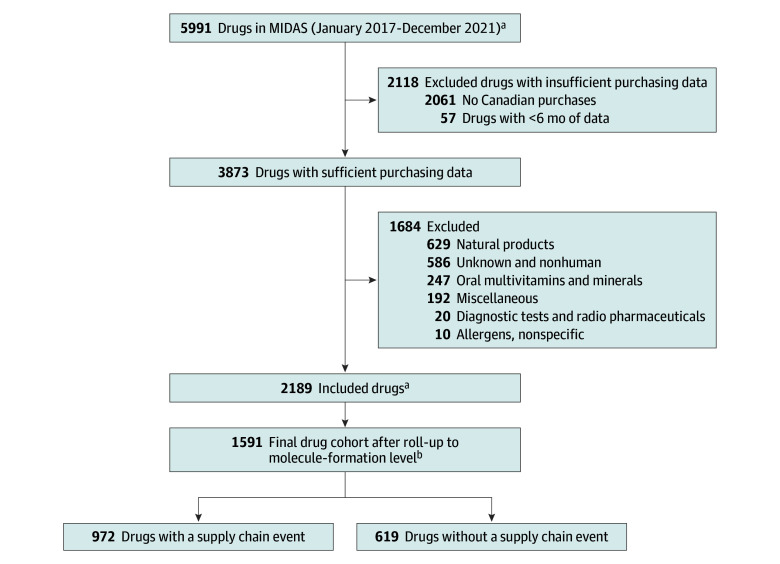
Cohort Selection Diagram ^a^Drugs were defined at the Anatomical Therapeutic Class Level 3 (ATC3) molecule list new form code 3 level. ^b^Drugs were aggregated from the ATC3 molecule list new form code 3 level to unique molecule-formulation level. Products with the same molecule but different new form codes that belonged to the same formulation category were combined; no drugs were excluded at this step.

After exclusions, 1591 drug forms remained in the final drug cohort (Figure 1), and 972 (61%) were exposed to 1919 supply chain issue events from 2017 to 2021. All events were EDS-matched to 10 comparators each to give a total of 19 190 comparators.

The supply chain issue group had more drugs older than 20 years (1373 drugs [72%] vs 11 305 drugs [59%]), generic drugs (1669 drugs [87%] vs 14 405 drugs [75%]), drugs with 2 to 4 manufacturers (847 drugs [44%] vs 5339 drugs [28%]) and drugs with previous supply chain issues (1666 drugs [87%] vs 5806 drugs [30%]). In the supply chain issue group, there were fewer drugs with 1 manufacturer compared with the control group (738 drugs [38%] vs 11 434 drugs [60%]) (Table). The most common supply chain issue was a shortage (1471 drugs [77%]), the most common duration was less than 6 months (1224 drugs [64%]), and the most common reason was disruption in manufacturing (931 drugs [49%]) (eTable 4 in Supplement 1).

**Table.** Characteristics of Drugs With Supply Chain Issue Events vs Matched Comparators for Incident Shortage Analysis, 2017-2021^a^

Variables	Drugs, No. (%)	
	Supply chain issue events (n = 1919)	Matched controls (n = 19 190)
Anatomical therapeutic chemical class level 1		
Alimentary tract and metabolism	159 (8)	1966 (10)
Blood	114 (6)	710 (4)
Cardiovascular	229 (12)	1818 (9)
Dermatologicals	115 (6)	1538 (8)
Genitourinary and sex hormones	149 (8)	981 (5)
Systemic hormonal	63 (3)	479 (2)
Anti-infective	243 (13)	2267 (12)
Antineoplastic and immunomodulating	173 (9)	2854 (15)
Musculoskeletal	73 (4)	735 (4)
Nervous	328 (17)	2966 (15)
Antiparasitic & insecticides	16 (1)	229 (1)
Respiratory	104 (5)	1365 (7)
Sensory	104 (5)	1075 (6)
Various	49 (3)	207 (1)
Formulation		
Inhaled	54 (3)	675 (3)
Ophthalmic or otic	94 (5)	978 (5)
Oral	962 (50)	9451 (49)
Parenteral	605 (32)	5581 (29)
Rectal	14 (1)	95 (1)
Topical	145 (8)	2085 (11)
Transdermal	20 (20)	138 (1)
Vaginal	25 (1)	187 (1)
Unit price, $		
<0.50	635 (33)	6865 (36)
0.50-0.99	233 (12)	1749 (9)
1.00-4.99	412 (21)	3251 (17)
5.00-19.99	278 (14)	2391 (12)
20.00-99.99	226 (12)	2536 (13)
≥100.00	135 (7)	2398 (12)
Age, y		
<5	72 (4)	1693 (9)
5-9	130 (7)	2266 (12)
10-19	344 (18)	3926 (20)
≥20	1373 (72)	11 305 (59)
Manufacturers, No.		
1	738 (38)	11 434 (60)
2-4	847 (44)	5339 (28)
5-9	250 (13)	1417 (7)
≥10	84 (4)	1000 (5)
Sales, $		
<100 000	188 (10)	2548 (13)
100 000 to 499 999	421 (22)	4300 (22)
500 000 to 1.9 million	527 (27)	4605 (24)
2.0 million to 4.9 million	351 (18)	3057 (16)
≥5.0 million	432 (23)	4680 (24)
World Health Organization essential medicine		
No	1191 (62)	13 125 (68)
Yes	728 (38)	6065 (32)
Therapeutic equivalent		
No	552 (29)	5476 (29)
Yes	1367 (71)	13 714 (71)
Active pharmaceutical ingredients, No.		
1	1617 (84)	15 327 (80)
2	225 (12)	2851 (15)
3	40 (2)	644 (3)
≥4	37 (37)	368 (2)
DINs, No.		
1	259 (13)	5115 (27)
2-3	442 (23)	5543 (29)
4-9	710 (37)	5082 (26)
10-24	369 (19)	1989 (10)
≥25	139 (7)	1461 (8)
Ontario drug benefit coverage, % of DINs		
0	667 (35)	7927 (41)
1-49	296 (15)	1887 (10)
50-99	539 (28)	4048 (21)
100	417 (22)	5328 (28)
Schedule		
Ethical	135 (7)	765 (4)
Targeted or narcotic	121 (4)	653 (3)
Over the counter	91 (5)	2837 (15)
Prescription	1431 (75)	12 780 (67)
Schedule D	117 (6)	1998 (10)
Schedule G	24 (1)	157 (1)
Tier 3		
No	1771 (92)	18 474 (96)
Yes	148 (8)	716 (4)
Dominant sector		
Hospital	1324 (69)	14 112 (74)
Retail	449 (23)	3510 (18)
Both	146 (8)	1568 (8)
Brand		
No	1669 (87)	14 405 (75)
Yes	250 (13)	4785 (25)
Previous supply chain issue		
No	253 (13)	13 384 (70)
Yes	1666 (87)	5806 (30)

Abbreviation: DIN, drug identification number.

^a^ Defined as at least a 33% decrease in purchases within 6 months following the supply chain issue compared with the 3 months prior.

### Incident Shortages and Associated Characteristics

**Figure 2.**
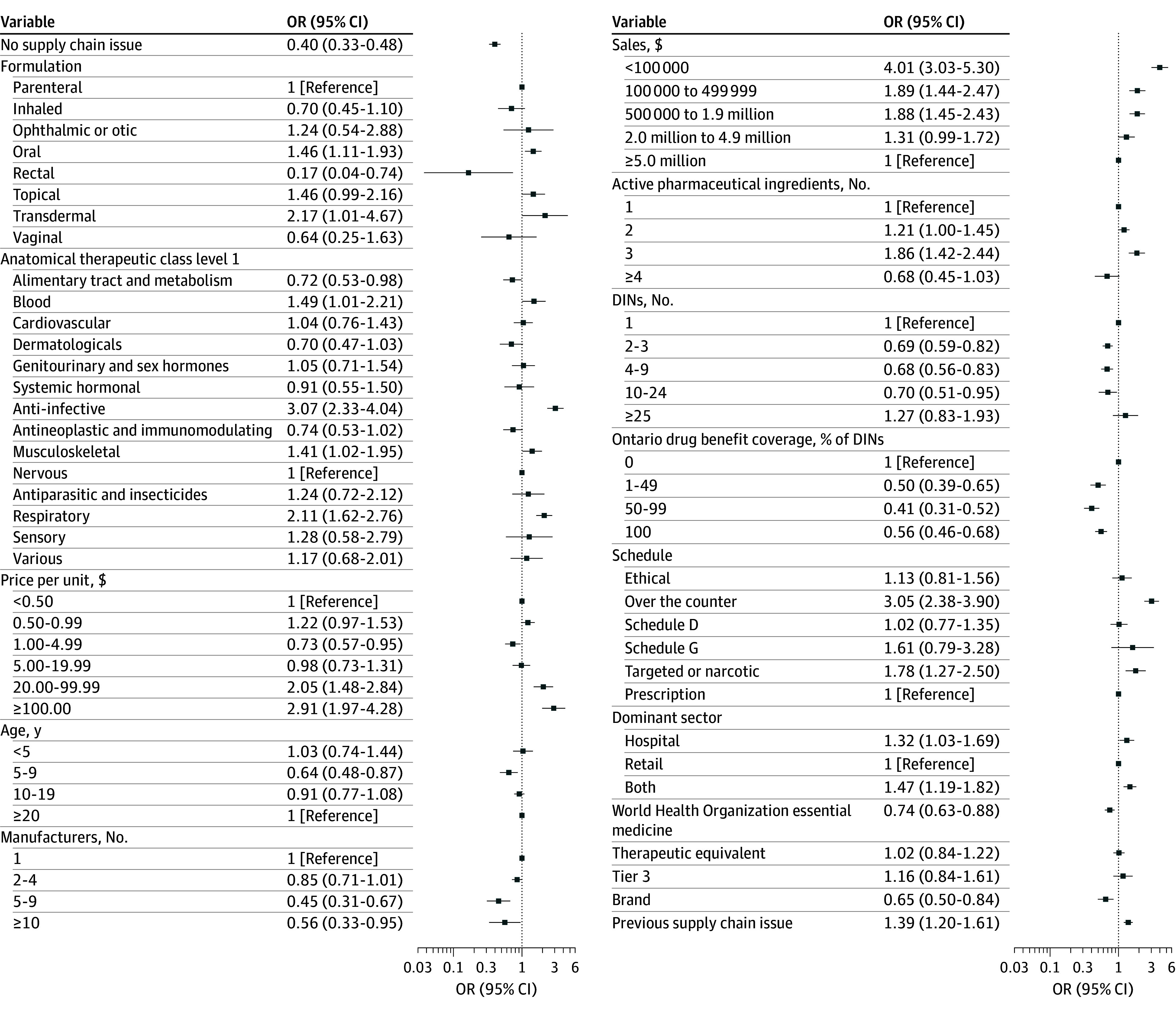
Forest Plot of Odds Ratios (ORs) for Incident Shortage Outcome Defined as at least a 33% decrease in purchases within 6 months following the supply chain issue compared with the 3 months prior. DIN indicates drug identification number.

Of the 1919 supply chain issue events, 216 (11%) had an incident shortage within 6 months, compared with 1271 comparators (7%) (eTable 5 in Supplement 1). Adjusting for drug characteristics, the odds of incident shortage for comparison drugs was lower than drugs with supply chain issues (odds ratio [OR], 0.40; 95% CI, 0.33-0.48) (Figure 2).

Factors with the lowest odds of incident shortage were rectal formulation vs parenteral formulations (OR, 0.17; 95% CI, 0.04-0.74), ODB coverage for 50% to 99% of DINs vs no coverage (OR, 0.41; 95% CI, 0.31-0.52), and 5 to 9 manufacturers vs 1 manufacturer (OR, 0.45; 95% CI, 0.31-0.67). Other characteristics with significantly decreased odds of incident shortage were alimentary tract and metabolism ATC class; unit price of $1.00 to $4.99; age of 5 to 9 years; more than 10 manufacturers; WHO essential medicines; having 2 to 3, 4 to 9, and 10 to 24 DINs; ODB coverage for 1% to 49% and 100% of DINs; and brand drugs (Figure 2).

Factors with the highest odds of incident shortage were baseline sales less than $100 000 vs sales greater than $5 million (OR, 4.01; 95% CI, 3.03-5.30), anti-infective ATC class vs nervous ATC class (OR, 3.07; 95% CI, 2.33-4.04), and OTC drugs vs prescription (OR, 3.05; 95% CI, 2.38-3.90) (Figure 2). Other covariates with significantly high odds of incident shortage were blood, musculoskeletal, and respiratory ATC classes; oral and transdermal formulations; unit prices greater than $20; baseline sales of $100 000 to 499 999 and $500 000 to $1 999 999; having 2 and 3 APIs; targeted and narcotic drugs; having previous supply chain issues; and dominant sector being hospital or both hospital and retail (Figure 2).

Severe incident shortages occurred in 100 supply chain issues (5%) and 429 comparators (2%) (eTable 5 in Supplement 1). Adjusting for drug characteristics, the odds of severe incident shortages was lower for comparators vs supply chain issues (OR, 0.31; 95% CI, 0.23-0.41) (eFigure 3 in Supplement 1).

Characteristics associated with severe incident shortages were consistent with the primary shortage analysis. Covariates with lowest odds of severe incident shortages were 5 to 9 manufacturers vs 1 manufacturer (OR, 0.03; 95% CI, 0.00-0.19), more than 10 manufacturers vs 1 manufacturer (OR, 0.23; 95% CI, 0.07-0.69), and age of 5 to 9 years vs 20 or more years (OR, 0.45; 95% CI, 0.26-0.77) (eFigure 3 in Supplement 1). Other factors with significantly low odds of a severe incident shortage were antineoplastic and immunomodulating ATC classes, 2 to 4 manufacturers, 4 APIs, 2 to 3 DINs, ODB coverage for 50% to 99% of DINs, and brand drugs.

Characteristics with the greatest odds of severe incident shortage were baseline sales less than $100 000 vs sales greater than $5 million (OR, 5.08; 95% CI, 3.27-7.87), transdermal formulation vs parenteral formulations (OR, 4.16; 95% CI, 1.58-10.97), and unit price greater than $100 vs less than $0.50 (OR, 3.19; 95% CI, 1.73-5.86) (eFigure 3 in Supplement 1). Other factors significantly associated with severe incident shortages were anti-infective and respiratory ATC classes, oral and topical formulations, 2 and 3 APIs, OTC drugs, schedule D drugs, targeted and narcotic drugs, a previous supply chain issue, hospital dominant sector or both hospital and retail.

### Shortage Intensity and Associated Characteristics

**Figure 3.**
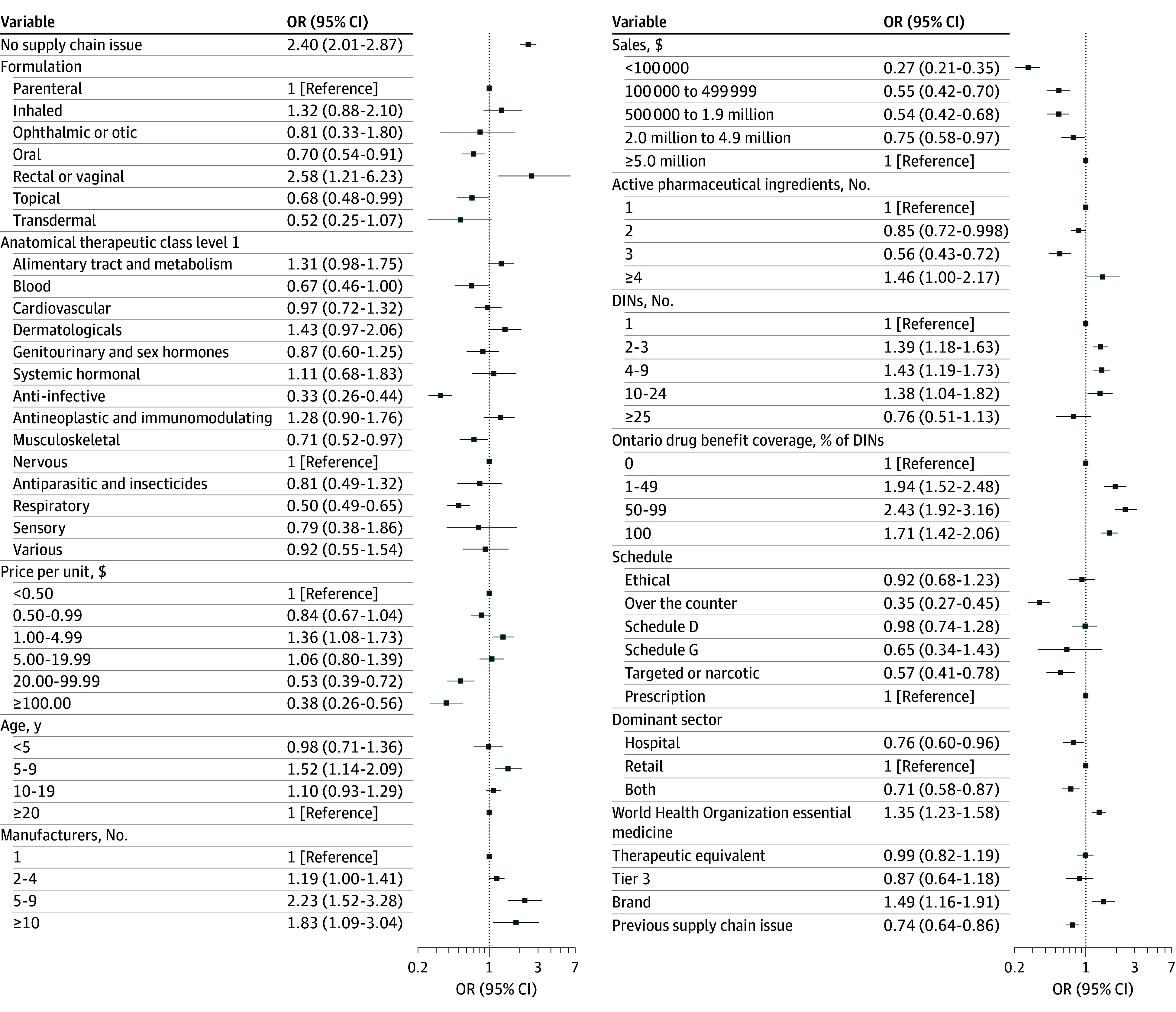
Forest Plot of Odds Ratios (ORs) for Shortage Intensity Scores Equal to Zero DIN indicates drug identification number.

**Figure 4.**
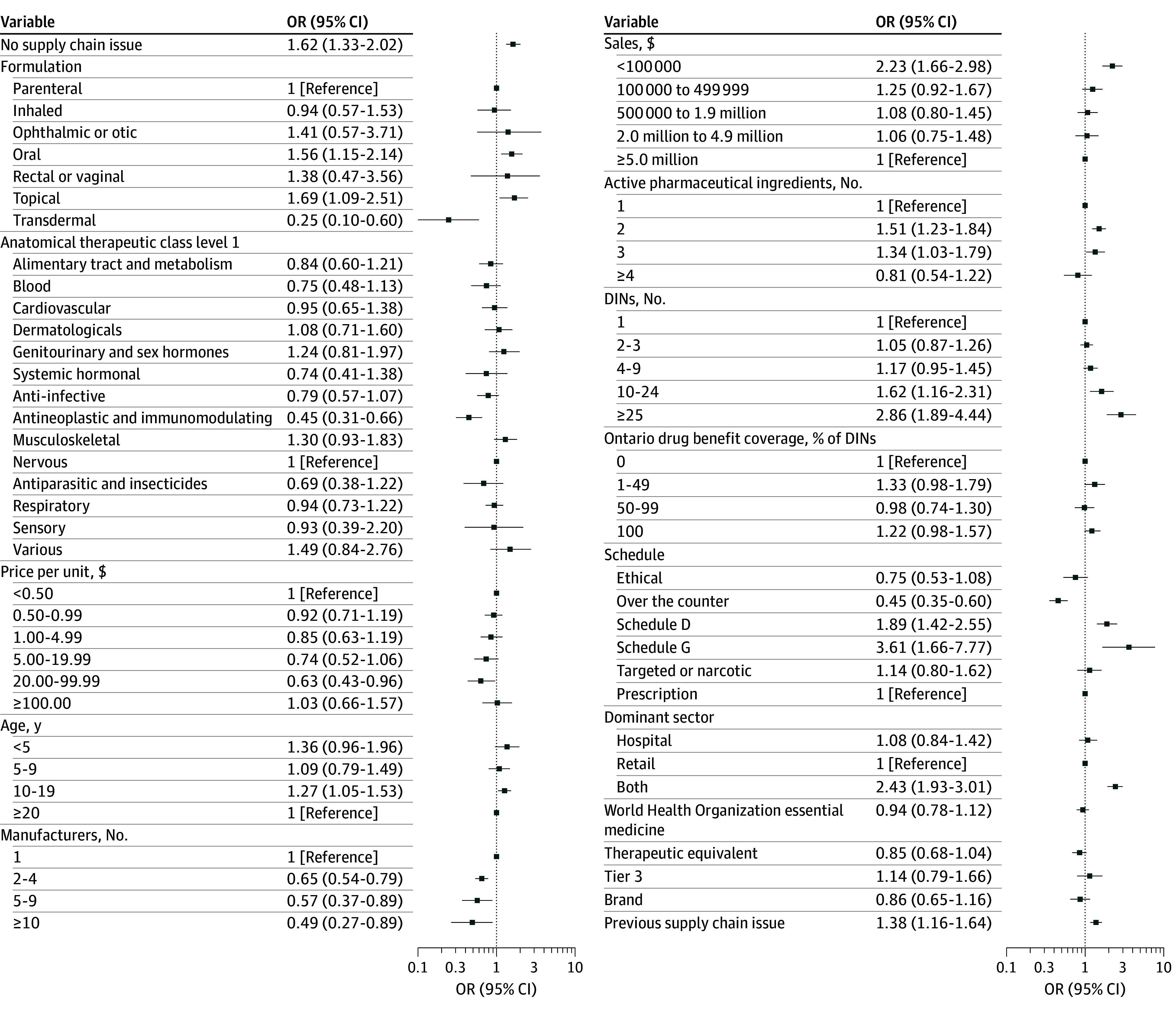
Forest Plot of Odds Ratios for Nonzero Shortage Intensity Scores Ranging From Greater Than 0 to 1 DIN indicates drug identification number.

For the secondary outcome, adjusting for other drug characteristics, the odds of having a shortage intensity of 0 for comparison drugs was greater than drugs with supply chain issues (OR, 2.40; 95% CI, 2.01-2.87) (Figure 3). The odds of having a higher intensity shortage for comparison drugs was higher compared with drugs with reported supply chain issues (OR, 1.62; 95% CI, 1.33-2.02) (Figure 4), indicating that comparison drugs rarely faced decreases in purchases, but when they did it was of high intensity; this was due to drugs with strong seasonal fluctuations, such as antibiotics.

Factors with the largest magnitude of association with a shortage intensity of 0 were rectal and vaginal formulations vs parenteral (OR, 2.58; 95% CI, 1.21-6.23), ODB coverage for 50% to 99% of DINs vs no coverage (OR, 2.43; 95% CI, 1.92-3.16), and 5 to 9 manufacturers vs 1 manufacturer (OR, 2.23; 95% CI, 1.52-3.28) (Figure 3). Other factors significantly associated with a 0 intensity were unit price of $1.00 to $4.99, age of 5 to 9 years, more than 10 manufacturers, WHO essential medicine, 2 to 24 DINs, public drug coverage for 1% to 49% of DINs and 100% of DINs, and being a brand.

Factors with the largest magnitude of association with higher shortage intensity were schedule G drugs vs prescription drugs (controlled drugs; OR, 3.61; 95% CI, 1.66-7.77), more than 25 DINs available vs 1 DIN (OR, 2.86; 95% CI, 1.89-4.44), and dominant sector of both retail and hospital vs retail dominant (OR, 2.43; 95% CI, 1.93-3.01) (Figure 4). Other drug characteristics significantly associated with a high intensity were oral and topical formulations, age of 10 to 19 years, baseline sales less than $100 000, 2 and 3 APIs, 10 to 24 DINs, schedule D drugs (biologics), and a previous shortage.

### Sensitivity Analysis

Sensitivity analyses of incident shortages were consistent with the main results (eTables 6-8 and eFigures 4-8 in Supplement 1). For supply chain issue exposure, excluding discontinuations yielded an OR of 0.40 (95% CI, 0.33-0.49), using 5 controls produced an OR of 0.34 (95% CI, 0.28-0.43), and extending follow-up to 9 months resulted in an OR of 0.39 (95% CI, 0.33-0.47). The post–COVID-19 pandemic flag was not significant (OR, 0.92; 95% CI, 0.79-1.07) and was excluded from the model. For shortage intensity, the multivariate regression results were consistent with the main results. No supply chain issue was associated with a significantly lower odds of a low (OR, 0.35; 95% CI, 0.27-0.46), medium (OR, 0.35; 95% CI, 0.25-0.48), or high (OR, 0.59; 95% CI, 0.43-0.82) shortage intensity compared with no shortage (intensity = 0). When there was a shortage, experiencing a high intensity shortage was more likely than a low intensity shortage, similar to the main results.

## Discussion

In this cohort study, we found that 9 in 10 supply chain issues did not experience an incident shortage, and 19 in 20 events did not face a severe incident shortage. These findings suggest that a supply chain issue alone does not translate into a population-level shortage affecting patients’ access to medications. Our study also highlights factors associated with occurrence and intensity of shortages, which can be used to anticipate shortage risk of drugs.

Many Canadian studies have examined drug shortage reports,^16,17,18^ and similar work has been conducted globally.^37,38,39,40,41^ However, our results highlight that manufacturer-level reports do not reflect population-level drug availability, and solely examining these reports fails to capture true shortages. Our study uniquely examines the occurrence of actual shortages, similar to the Patented Medicine Prices Review Board in Canada report, which assessed shortage report impact on public beneficiaries.^42^ Our study builds upon this work by modeling actual shortages to ascertain factors and measuring shortage intensity. Although shortage reporting is essential, it may lead to many false-positive reports when there is no actual shortage in available supply, particularly given the 5-day time frame manufacturers are required to report within; this emphasizes the need for a unified shortage definition that ensures surveillance efforts target clinically relevant events.

Our study identifies drug characteristics associated with occurrence and the intensity of shortages. Our results suggest that drugs with public plan coverage and multiple manufacturers were more protected from shortages. Conversely, drugs with small market sizes and oral formulations are especially vulnerable to severe shortages. Findings suggest that having multiple DINs offers protection against shortages, although when they do occur, they are high intensity; this was due to unique supply chain events, such as the ranitidine recall, where all drug products were affected. OTC drugs have higher shortage occurrence but lower intensity; however, reporting of OTC supply disruptions are not mandated which could bias reporting toward more serious events.

In the US, 13.7% of supply issues faced a shortage compared with 4.1% of controls, similarly indicating that not all supply issues lead to actual shortages.^19^ Similar to our results, in the US, parenteral medicines and WHO essential medicines were less likely to experience shortages.^19^ However, brand drugs were more likely to be associated with significant decreases.^19^ Shortage risk was greater for sole-sourced drugs similar to our findings, but lower for Canadian Tier 3 medicines which was nonsignificant in our model.^29^

Factors associated with shortage that were identified in our study can be leveraged to anticipate shortage risk across Canadian drugs. Supplementing these factors with clinical risk factors can inform the development of a national at-risk medicines list, incorporating both supply chain and clinical considerations. This risk-informed framework can help identify high-risk medicines, enabling policymakers to prioritize interventions and tailor policies to each drug’s risk profile. The findings from this study have helped develop Health Canada’s Critical Vulnerable Drug list.^43^ Implementing targeted, evidence-informed strategies can mitigate supply disruptions, protect the drug supply, and improve patient outcomes.

### Limitations

Our study has limitations that need to be considered. Drug purchases do not necessarily reflect patient-level drug use and serve only as a proxy for actual drug utilization. Moreover, the MIDAS dataset does not capture OTC purchases in nonpharmacy outlets and grocery stores, providing an incomplete view of OTC purchases.^4^ Factors such as unexpected market demand, regulatory changes, natural disasters, or economic conditions could not be included as covariates due to data limitations and may represent residual confounding. We did not evaluate whether specific supply chain reasons were associated with shortages due to inconsistencies in manufacturer-reported reasons, which represents an important area for future research. Differences in regulatory frameworks, reporting requirements, and market dynamics may limit generalizability to other countries. Additionally, the use of national-level data limits applicability to provincial or local settings within Canada. Because this analysis reflects a specific time window, the model requires updating to remain relevant to the current supply chain and policy landscape. However, the observed findings can be applicable to jurisdictions with similar reporting systems and market structures.

## Conclusions

In this cohort study of shortage incidence following Canadian supply chain events, most supply chain disruptions did not result in population-level drug shortages, emphasizing the distinction between upstream events and true shortages affecting availability. Setting a standardized threshold for shortages can ensure that surveillance efforts target clinically relevant events. A data-driven at-risk medicines framework can enhance early detection, improve preparedness, and guide evidence-based policies, safeguarding medication access in Canada.

## Notes

Supplement 1. eTable 1. Description of All Data Sources Used eTable 2. Definitions of Variables Included in Model eFigure 1. Definition of 33% Decrease Incident Shortage and 66% Decrease Severe Incident Shortage Outcome eTable 3. Specificity of Meaningful Decrease Thresholds eFigure 2. Definition of Shortage Intensity Outcome eTable 4. Characteristics of Drug Supply Chain Issue Events eTable 5. Proportion of Supply Chain Events and Comparators With Incident Shortage and Severe Incident Shortage eFigure 3. Forest Plot of Odds Ratios for Severe Incident Shortage ≥66% eTable 6. Proportion of Supply Chain Events and Comparators With Incident Shortage for Sensitivity Analyses eTable 7. Proportion of Supply Chain Events and Comparators With Severe Incident Shortage for Sensitivity Analyses eTable 8. Odds Ratios for Multivariate Regression Sensitivity Analysis for Shortage Intensity Outcome eFigure 4. Limited to Shortage-Events Excluding Discontinuations Sensitivity Analysis for Incident Shortage Outcome eFigure 5. Limited Number of Controls to 5 instead of 10 Sensitivity Analysis for Incident Shortage Outcome eFigure 6. Defining the Outcome Within 9 months of the Supply Chain Event Instead of 6 Months Sensitivity Analysis for Incident Shortage Outcome eFigure 7. Including the Post-COVID Pandemic Variable Sensitivity Analysis for Incident Shortage Outcome eFigure 8. Multivariate Regression Sensitivity Analysis for Shortage Intensity Outcome eReferences.

Supplement 2. Data Sharing Statement
