# Supplementary material for: Supply Chain Events and Risk of Drug Shortage in Canada
Source: JAMA Netw Open. 2026 Jun 4;9(6):e2616632. doi: 10.1001/jamanetworkopen.2026.16632 (PMC13237616; doi:10.1001/jamanetworkopen.2026.16632)
Supplement: Supplement 1. — eTable 1. Description of All Data Sources Used eTable 2. Definitions of Variables Included in Model eFigure 1. Definition of 33% Decrease Incident Shortage and 66% Decrease Severe Incident Shortage Outcome eTable 3. Specificity of Meaningful Decrease Thresholds eFigure 2. Definition of Shortage Intensity Outcome eTable 4. Characteristics of Drug Supply Chain Issue Events eTable 5. Proportion of Supply Chain Events and Comparators With Incident Shortage and Severe Incident Shortage eFigure 3. Forest Plot of Odds Ratios for Severe Incident Shortage ≥66% eTable 6. Proportion of Supply Chain Events and Comparators With Incident Shortage for Sensitivity Analyses eTable 7. Proportion of Supply Chain Events and Comparators With Severe Incident Shortage for Sensitivity Analyses eTable 8. Odds Ratios for Multivariate Regression Sensitivity Analysis for Shortage Intensity Outcome eFigure 4. Limited to Shortage-Events Excluding Discontinuations Sensitivity Analysis for Incident Shortage Outcome eFigure 5. Limited Number of Controls to 5 instead of 10 Sensitivity Analysis for Incident Shortage Outcome eFigure 6. Defining the Outcome Within 9 months of the Supply Chain Event Instead of 6 Months Sensitivity Analysis for Incident Shortage Outcome eFigure 7. Including the Post-COVID Pandemic Variable Sensitivity Analysis for Incident Shortage Outcome eFigure 8. Multivariate Regression Sensitivity Analysis for Shortage Intensity Outcome eReferences. [file jamanetwopen-e2616632-s001.pdf]

Supplemental Online Content

Santhireswaran A, Ho MKH, Kim KC, et al. Supply chain events and risk of drug shortage in Canada. *JAMA Netw. Open*. 2026;9(6):e2616632.
doi:10.1001/jamanetworkopen.2026.16632

eTable 1. Description of All Data Sources Used
eTable 2. Definitions of Variables Included in Model
eFigure 1. Definition of 33% Decrease Incident Shortage and 66% Decrease Severe Incident Shortage Outcome
eTable 3. Specificity of Meaningful Decrease Thresholds
eFigure 2. Definition of Shortage Intensity Outcome
eTable 4. Characteristics of Drug Supply Chain Issue Events
eTable 5. Proportion of Supply Chain Events and Comparators With Incident Shortage and Severe Incident Shortage
eFigure 3. Forest Plot of Odds Ratios for Severe Incident Shortage ≥66%
eTable 6. Proportion of Supply Chain Events and Comparators With Incident Shortage for Sensitivity Analyses
eTable 7. Proportion of Supply Chain Events and Comparators With Severe Incident Shortage for Sensitivity Analyses
eTable 8. Odds Ratios for Multivariate Regression Sensitivity Analysis for Shortage Intensity Outcome
eFigure 4. Limited to Shortage-Events Excluding Discontinuations Sensitivity Analysis for Incident Shortage Outcome
eFigure 5. Limited Number of Controls to 5 instead of 10 Sensitivity Analysis for Incident Shortage Outcome
eFigure 6. Defining the Outcome Within 9 months of the Supply Chain Event Instead of 6 Months Sensitivity Analysis for Incident Shortage Outcome
eFigure 7. Including the Post-COVID Pandemic Variable Sensitivity Analysis for Incident Shortage Outcome
eFigure 8. Multivariate Regression Sensitivity Analysis for Shortage Intensity Outcome
eReferences.

This supplemental material has been provided by the authors to give readers additional information about their work.

© 2026 Santhireswaran A et al. *JAMA Network Open*.

eTable 1: Description of all Data Sources used

Data Source	Description	Variables Used
**IQVIA Multinational Integrated Data Analysis (MIDAS)**	Monthly drug sales volume, sales value, and number of manufacturers for non-retail prescription and over-the-counter purchases made by pharmacies from wholesalers and manufacturers for 2017-2021.	Sales volume data for outcome, ATC, formulation, unit price, manufacturers, sales value, dominant sector
**Health Canada Drug Product Database (DPD)**	Characteristics of drugs marketed in Canada during 2017-2021	Age, number of active pharmaceutical ingredients (APIs), number of DINs, schedule
**Drug Shortages Canada**	Supply chain reports made by market authorization holders (manufacturers) between March 2017 – October 2021. This includes shortages for various reasons (including resulting from recalls, natural disasters, regulatory changes, business decisions, increased market demand) and product discontinuations.	Supply chain issue exposure, previous supply chain issue, supply chain issue characteristics (status, duration, etc.)
**World Health Organization (WHO) Essential Medicines List**	WHO full electronic eEML database downloaded in June 2022	WHO Essential Medicine
**ICES Drug List**	List of DINs covered by Ontario Drug Benefit (ODB)	Proportion of DINs covered by Ontario Drug Benefit (ODB). (ODB coverage was used as a predictor of public plan coverage in Canada because of data availability and Ontario being the largest province in Canada.[1])
**Health Canada Tier-3 Drug List**	Historical Tier-3 list from Health Canada and Health Canada Tier-3 website	Tier 3 drug

ATC: anatomical therapeutic chemical, API: active pharmaceutical ingredient, DIN: drug identification number

© 2026 Santhireswaran A et al. *JAMA Network Open*.

eTable 2: Definitions of Variables Included in Model

Variable	Definition and Sources
**Anatomical Therapeutic Chemical (ATC)**	Anatomical Therapeutic Chemical Level 1 classification from MIDAS.
**Formulation**	Use New Form Code (NFC) variable in MIDAS to assign drugs into the following formulation categories: parenteral, oral, ophthalmic/otic, inhaled, topical, transdermal, vaginal, rectal.
**Unit Price**	Average unit price of standardized units (SU) across study period. Calculated from total sales and total SU purchases in study period from MIDAS, reported in USD.
**Age**	Years since first marketed date from Drug Product Database (DPD).
**Manufacturers**	Calculated in the 6-month period prior to each drug's index date using the manufacturer variable in MIDAS. "REPACKAGER" was counted as one manufacturer.
**Sales**	Calculated average sales in the 6-month period prior to each drug's index date using sales data in MIDAS, reported in USD.
**WHO Essential Medicine**	Assigned using the WHO full electronic eEML database, downloaded in June 2022.
**Therapeutic Equivalent**	Assigned using a manual flag of drugs with an available clinical alternative in Canada.
**Number of APIs**	Number of active pharmaceutical ingredients as listed in DPD.
**Number of DINs**	Number of marketed DINs for each ingredient-formulation in the study period. DINs are a unique set of 8 numbers that are assigned to unique pharmaceutical products.
**Proportion of DINs Covered by ODB**	Calculated proportion of marketed DINs covered by Ontario Drug Benefit (ODB)[2] using ICES drug list.
**Schedule**	Drug categories assigned to products according to the *Food and Drug Regulations* and the *Controlled Drugs and Substances Act* from DPD.
**Tier 3**	Assigned using historical Tier-3 list from Health Canada, Tier 3 Health Canada website, and Tier 3 variable from Drug Shortages Canada reports. Tier-3 status is assigned to drug that when in shortage have the greatest potential impact on Canada's drug supply and healthcare system.
**Dominant Sector**	The sector (retail or hospital) that contributed 66% or more to the overall market of each drug. If neither sector contributed more than 66%, the category of "both" was assigned. Average in the 6-month period prior to each drug's index date was calculated using MIDAS data at the sector level.
**Brand vs Generic**	Assigned using a manual flag of brand drugs.
**Previous Supply Chain Issue**	Flagged drugs with a previous supply issue report since March 2017 using Drug Shortages Canada.

© 2026 Santhireswaran A et al. *JAMA Network Open*.

eFigure 1: Definition of 33% Decrease Incident Shortage and 66% Decrease Severe Incident Shortage Outcome

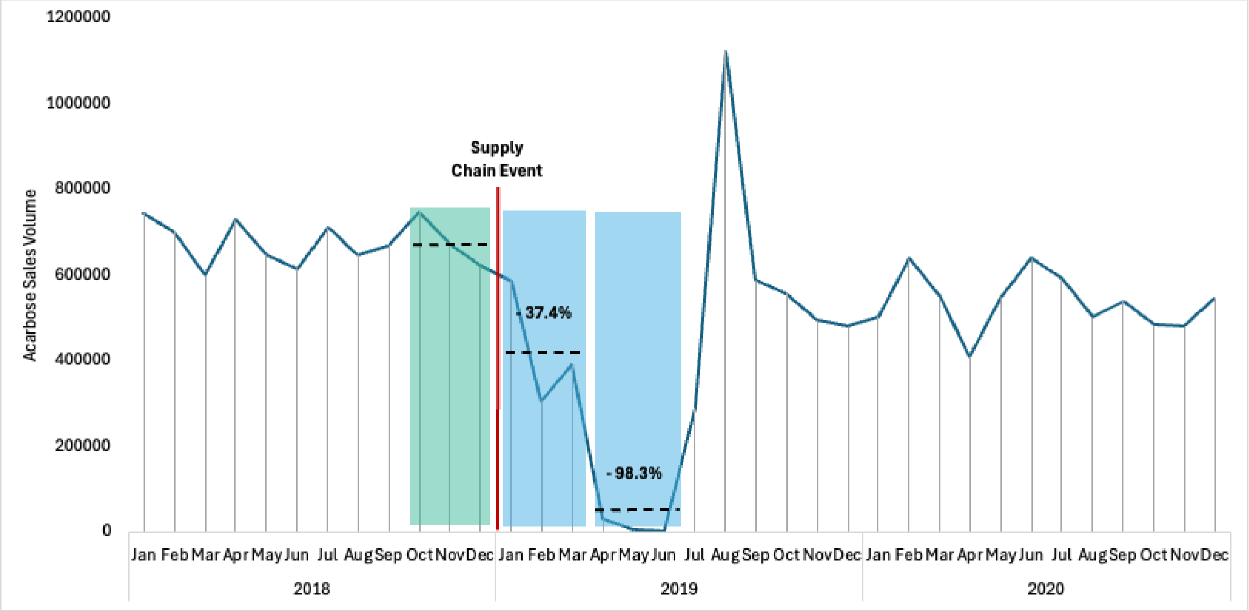

In this case example, oral acarbose had a supply chain issue in January 2019. The quarter following the exposure has a 37.4% decrease in sales volume compared to the quarter prior to the supply chain issue, and the second quarter following the supply chain report had a 98.3% decrease. Therefore, this case will be flagged as having a 33% decrease incident shortage and a 66% decrease severe incident shortage. This definition has been used in prior literature.[3-5]

eTable 3: Sensitivity and Specificity of Meaningful Decrease Thresholds

	**≥5%**	**≥10%**	**≥20%**	**≥33%**	**≥50%**
**Sensitivity**	100%	58.7%	31.8%	16.8%	10.3%
**Specificity**	0	47.8%	78.4%	90.5%	96.1%

To evaluate the appropriateness of the ≥33% decrease threshold for defining shortages, we scanned national purchasing data using 3-month moving averages to identify decreases of varying magnitudes (≥5%, ≥10%, ≥20%, ≥33%, ≥50%). For each identified decrease, we assessed whether a supply chain issue report occurred within the preceding 6 months. Sensitivity was calculated as the proportion of supply chain issue reports associated with a decrease at or above the specified threshold (true positives / [true positives + false negatives]), and specificity was calculated as the proportion of observations without a supply chain issue report that did not experience a decrease at or above the threshold (true negatives / [true negatives + false positives]). The ≥33% threshold was selected as it provided a balance between sensitivity and specificity while capturing substantial reductions in purchasing consistent with meaningful shortages.

© 2026 Santhireswaran A et al. *JAMA Network Open.*

eFigure 2: Definition of Shortage Intensity Outcome

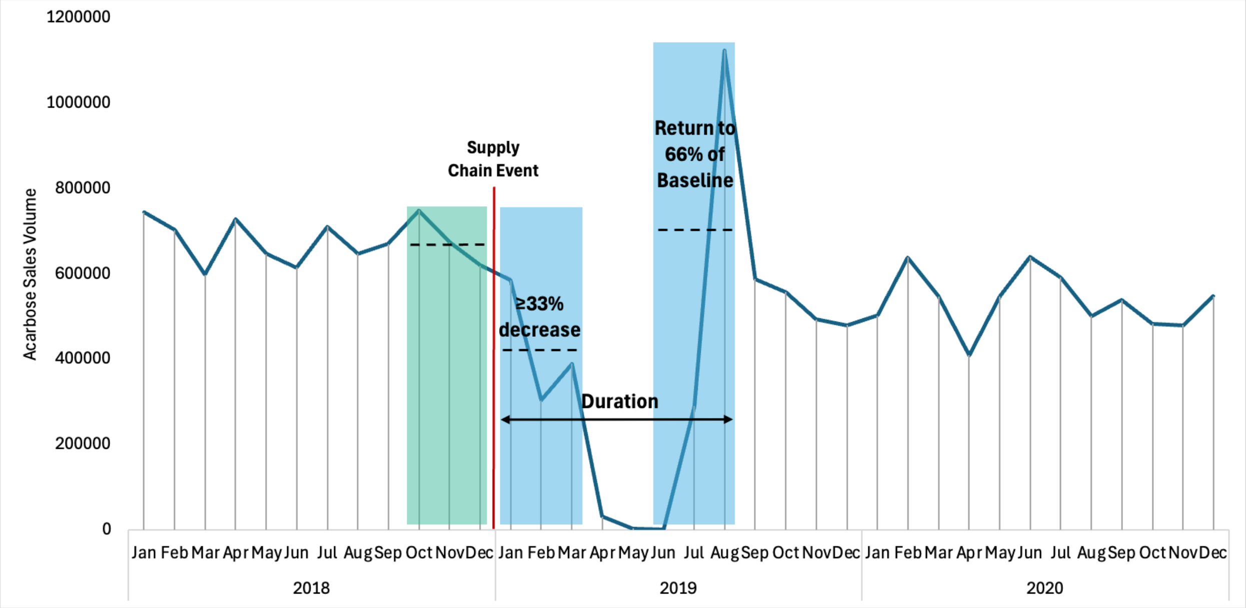

The secondary outcome was a continuous shortage intensity measure defined as the reduction in drug purchases multiplied by duration of the decrease. The reduction was the percent difference between the total purchase volume in the quarter preceding the supply chain issue and the first rolling quarter sum that fell below 33% of the baseline. The shortage duration was measured in months from the quarter of the ≥33% decrease until the rolling quarterly sum recovered to at least 66% of pre-supply chain event sales volume. As shown in the figure, acarbose had an incident shortage (≥33% decrease) with a 37.4% decrease from the baseline in the moving quarterly sum starting in the first month following the supply chain issue. The sales volume returned to 66% of baseline levels in the moving quarterly sum starting in the seventh month following the supply chain issue, meaning the shortage duration was 6 months. Therefore, the raw shortage intensity score was 37.4 × 6 months = 224.4. Given this calculation the maximum intensity value was a 100% decrease over 24 months giving a maximum intensity of 2400. For ease in modelling the raw scale of 0-2400 was rescaled to a 0-1 scale. The final intensity score for this case example was 0.09. This definition has been used in previous literature.[6]

© 2026 Santhireswaran A et al. *JAMA Network Open*.

eTable 4: Characteristics of Drug Supply Chain Issue Events

**Type of Issue**	**N (%)**
Shortage	1471 (77)
Discontinuations	448 (23)
**Duration in months**	
<6	1224 (64)
6-11	284 (15)
12-23	225 (12)
24-35	72 (4)
>36	114 (6)
**Reason for supply chain issue[a]**	
Business reasons	173 (9)
Delay in shipping	320 (17)
Demand increase	182 (9)
Disruption in manufacturing	931 (49)
Good manufacturing practice issues	37 (2)
Shortage of active ingredient	33 (2)
Shortage of inactive ingredient	11 (1)
Other	232 (12)

[a] Reasons for supply chain issues are selected by market authorization holders at the time of submitting a supply chain issue report from a predefined list of options in the Drug Shortages Canada reporting system.

eTable 5: Proportion of Supply Chain Events and Comparators with Incident Shortage and Severe Incident Shortage

**Incident Shortage (≥33% decrease in sales volume)**		
	**No Incident Shortage (≥33%)**	**Incident Shortage (≥33%)**
**Exposed**	88.7% (1703)	11.3% (216)
**Unexposed**	93.4% (17919)	6.6% (1271)
**Severe Incident Shortage (≥66% decrease in sales volume)**		
	**No Severe Incident Shortage (≥66%)**	**Severe Incident Shortage (≥66%)**
**Exposed**	94.8% (1819)	5.2% (100)
**Unexposed**	97.8% (18761)	2.2% (429)

© 2026 Santhireswaran A et al. *JAMA Network Open.*

eFigure 3: Forest Plot of Odds Ratios for Severe Incident Shortage ≥66%

Variable	OR (95% CI)
No Supply Chain Issue	0.305 (0.228, 0.408)
**Formulation**	
Parenteral	1 (Reference)
Inhaled	0.995 (0.477, 2.076)
Ophthalmic/Otic	3.378 (0.664, 17.175)
Oral	2.145 (1.393, 3.302)
Rectal	0.692 (0.081, 5.934)
Topical	2.844 (1.544, 5.239)
Transdermal	4.158 (1.576, 10.972)
Vaginal	0.639 (0.134, 3.062)
**Anatomical Therapeutic Class (ATC) Level–1**	
Alimentary tract & Metabolism	0.635 (0.376, 1.072)
Blood	1.512 (0.824, 2.773)
Cardiovascular	0.899 (0.527, 1.534)
Dermatologicals	0.581 (0.31, 1.091)
Genitourinary & Sex hormones	1.411 (0.804, 2.477)
Systemic hormonal	0.533 (0.2, 1.425)
Antiinfective	2.437 (1.57, 3.783)
Antineoplastic & Immunomodulating	0.467 (0.271, 0.805)
Musculoskeletal	1.328 (0.786, 2.245)
Nervous	1 (Reference)
Antiparasitic & Insecticides	0.537 (0.19, 1.519)
Respiratory	1.657 (1.076, 2.553)
Sensory	0.582 (0.123, 2.749)
Various	0.779 (0.305, 1.995)
**Unit Price**	
Less than $0.50/unit	1 (Reference)
$0.50 – $0.99/unit	1.286 (0.902, 1.834)
$1 – $4.99/unit	0.671 (0.445, 1.011)
$5 – $19.99/unit	0.823 (0.509, 1.331)
$20 – $99.99/unit	1.452 (0.852, 2.474)
$100+/unit	3.188 (1.734, 5.861)
**Age**	
Less than 5 years	1.202 (0.716, 2.019)
5 – 9 years	0.451 (0.264, 0.771)
10 – 19 years	0.961 (0.725, 1.273)
20+ years	1 (Reference)
**Manufacturers**	
1 manufacturer	1 (Reference)
2 – 4 manufacturers	0.456 (0.334, 0.621)
5 – 9 manufacturers	0.025 (0.003, 0.186)
10+ manufacturers	0.225 (0.073, 0.694)
**Sales**	
Less than 100k sales	5.076 (3.272, 7.874)
100k – 499k sales	1.397 (0.899, 2.173)
500k – 1.9 million sales	1.174 (0.755, 1.827)
2 – 4.9 million sales	1.138 (0.694, 1.866)
5 million+ sales	1 (Reference)
**Number of Active Pharmaceutical Ingredients (APIs)**	
1 API	1 (Reference)
2 APIs	1.39 (1.037, 1.863)
3 APIs	2.147 (1.371, 3.362)
4+ APIs	0.474 (0.231, 0.973)
**Number of Drug Identification Numbers (DINs)**	
1 DIN	1 (Reference)
2 – 3 DINs	0.631 (0.483, 0.824)
4 – 9 DINs	0.743 (0.538, 1.024)
10 – 24 DINs	0.793 (0.46, 1.365)
> 25 DINs	1.037 (0.459, 2.342)
**Ontario Drug Benefit (ODB) Coverage**	
0% DINs Covered	1 (Reference)
1 – 49% DINs Covered	0.812 (0.526, 1.251)
50 – 99% DINs Covered	0.543 (0.355, 0.83)
100% DINs Covered	0.75 (0.549, 1.023)
**Schedule**	
Ethical	1.082 (0.639, 1.832)
Over–the–Counter	1.892 (1.244, 2.876)
Schedule D	1.693 (1.081, 2.652)
Schedule G	2.517 (0.912, 6.95)
Targeted/Narcotic	2.616 (1.587, 4.31)
Prescription	1 (Reference)
**Dominant Sector**	
Hospital	1.685 (1.14, 2.489)
Retail	1 (Reference)
Both	2.148 (1.591, 2.9)
World Health Organization (WHO) essential medicine	0.761 (0.578, 1.002)
Therapeutic equivalent	0.909 (0.674, 1.228)
Tier–3	0.913 (0.49, 1.699)
Brand	0.638 (0.421, 0.966)
Previous supply chain issue	1.899 (1.485, 2.429)

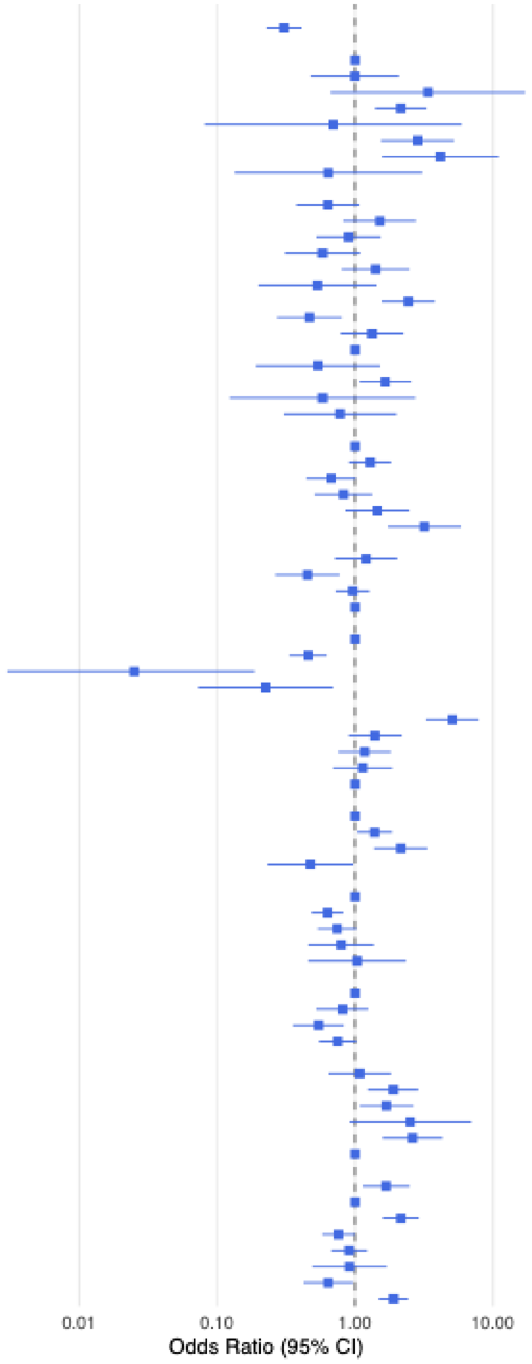

eTable 6: Proportion of Supply Chain Events and Comparators with Incident Shortage for Sensitivity Analyses

		Final Model	No Discontinuations	Cases matched with 5 Controls	Outcome in 9 months
**Exposed**	**No Incident Shortage (≥33%)**	88.7% (1,703)	88.8% (1,197)	88.7% (1,703)	87.9% (1,686)
	**Incident Shortage (≥33%)**	11.3% (216)	11.2% (151)	11.3% (216)	12.1% (233)
**Unexposed**	**No Incident Shortage (≥33%)**	93.4% (17,919)	93.9% (12,655)	94.0% (9,019)	92.7% (17,795)
	**Incident Shortage (≥33%)**	6.6% (1,271)	6.2% (825)	6.0% (576)	7.3% (1,395)

eTable 7: Proportion of Supply Chain Events and Comparators with Severe Incident Shortage for Sensitivity Analyses

		Final Model	No Discontinuations	Cases matched with 5 Controls	Outcome in 9 months
**Exposed**	**No Severe Incident Shortage (≥66%)**	94.8% (1,819)	95.0% (1,281)	94.8% (1,819)	94.1% (1,806)
	**Severe Incident Shortage (≥66%)**	5.2% (100)	5.0% (67)	5.2% (100)	5.9% (113)
**Unexposed**	**No Severe Incident Shortage (≥66%)**	97.8% (18,761)	97.5% (13,149)	97.8% (9,387)	97.5% (18,713)
	**Severe Incident Shortage (≥66%)**	2.2% (429)	2.5% (331)	2.2% (208)	2.5% (477)

eTable 8: Odds Ratios for Multivariate Regression Sensitivity Analysis for Shortage Intensity Outcome

Variable	Outcome Category	Odds Ratio	95% Lower CI	95% Upper CI
No Supply Chain Issue	0-0.1 vs 0	0.35	0.269	0.456
No Supply Chain Issue	0.1-0.4 vs 0	0.346	0.252	0.476
No Supply Chain Issue	>0.4 vs 0	0.591	0.425	0.82
Anatomical Therapeutic Class (ATC) Level-1 (Ref: Nervous)				
Alimentary tract & Metabolism	0-0.1 vs 0	0.732	0.485	1.104
Alimentary tract & Metabolism	0.1-0.4 vs 0	0.485	0.264	0.888
Alimentary tract & Metabolism	>0.4 vs 0	1.145	0.67	1.957
Blood	0-0.1 vs 0	0.755	0.415	1.374
Blood	0.1-0.4 vs 0	2.883	1.581	5.257
Blood	>0.4 vs 0	1.646	0.824	3.289
Cardiovascular	0-0.1 vs 0	0.803	0.505	1.278
Cardiovascular	0.1-0.4 vs 0	1.434	0.836	2.458
Cardiovascular	>0.4 vs 0	1.168	0.664	2.057
Dermatologicals	0-0.1 vs 0	0.65	0.371	1.139
Dermatologicals	0.1-0.4 vs 0	0.637	0.34	1.194
Dermatologicals	>0.4 vs 0	0.728	0.385	1.377
Genitourinary & Sex hormones	0-0.1 vs 0	0.866	0.491	1.527
Genitourinary & Sex hormones	0.1-0.4 vs 0	1.302	0.698	2.431
Genitourinary & Sex hormones	>0.4 vs 0	1.574	0.848	2.922
Systemic hormonal	0-0.1 vs 0	0.961	0.494	1.868
Systemic hormonal	0.1-0.4 vs 0	1.089	0.463	2.56
Systemic hormonal	>0.4 vs 0	0.654	0.224	1.914
Antiinfective	0-0.1 vs 0	3.004	2.059	4.383
Antiinfective	0.1-0.4 vs 0	2.718	1.671	4.421
Antiinfective	>0.4 vs 0	3.588	2.241	5.745
Antineoplastic & Immunomodulating	0-0.1 vs 0	0.829	0.525	1.308
Antineoplastic & Immunomodulating	0.1-0.4 vs 0	0.88	0.509	1.523
Antineoplastic & Immunomodulating	>0.4 vs 0	0.559	0.299	1.045
Musculoskeletal	0-0.1 vs 0	1.377	0.892	2.124
Musculoskeletal	0.1-0.4 vs 0	1.068	0.607	1.879
Musculoskeletal	>0.4 vs 0	1.82	1.052	3.148
Antiparasitic & Insecticides	0-0.1 vs 0	1.759	0.888	3.482
Antiparasitic & Insecticides	0.1-0.4 vs 0	0.253	0.058	1.109
Antiparasitic & Insecticides	>0.4 vs 0	2.071	0.896	4.786
Respiratory	0-0.1 vs 0	1.971	1.385	2.805

© 2026 Santhireswaran A et al. *JAMA Network Open*.

Respiratory	0.1-0.4 vs 0	1.735	1.108	2.718
Respiratory	>0.4 vs 0	2.247	1.402	3.601
Sensory	0-0.1 vs 0	1.49	0.538	4.129
Sensory	0.1-0.4 vs 0	0.984	0.219	4.427
Sensory	>0.4 vs 0	1.291	0.36	4.633
Various	0-0.1 vs 0	0.716	0.32	1.602
Various	0.1-0.4 vs 0	0.54	0.158	1.85
Various	>0.4 vs 0	2.855	1.32	6.178
Formulation (Ref: Parenteral)				
Inhaled	0-0.1 vs 0	0.731	0.414	1.292
Inhaled	0.1-0.4 vs 0	0.547	0.245	1.226
Inhaled	>0.4 vs 0	0.636	0.243	1.664
Ophthalmic/Otic	0-0.1 vs 0	0.676	0.223	2.048
Ophthalmic/Otic	0.1-0.4 vs 0	1.265	0.259	6.186
Ophthalmic/Otic	>0.4 vs 0	2.023	0.512	7.988
Oral	0-0.1 vs 0	0.879	0.586	1.319
Oral	0.1-0.4 vs 0	1.255	0.782	2.015
Oral	>0.4 vs 0	2.422	1.534	3.824
Rectal/Vaginal	0-0.1 vs 0	0.158	0.036	0.689
Rectal/Vaginal	0.1-0.4 vs 0	0.392	0.111	1.391
Rectal/Vaginal	>0.4 vs 0	1.186	0.329	4.274
Topical	0-0.1 vs 0	0.686	0.39	1.209
Topical	0.1-0.4 vs 0	1.577	0.823	3.024
Topical	>0.4 vs 0	3.863	2.068	7.216
Transdermal	0-0.1 vs 0	0.503	0.066	3.854
Transdermal	0.1-0.4 vs 0	5.469	2.195	13.624
Transdermal	>0.4 vs 0	0.532	0.107	2.634
Age (Ref: 20+ years)				
Less than 5 years	0-0.1 vs 0	0.874	0.537	1.423
Less than 5 years	0.1-0.4 vs 0	1.196	0.693	2.063
Less than 5 years	>0.4 vs 0	1.175	0.671	2.058
5 - 9 years	0-0.1 vs 0	0.75	0.505	1.115
5 - 9 years	0.1-0.4 vs 0	0.657	0.397	1.087
5 - 9 years	>0.4 vs 0	0.605	0.345	1.061
10 - 19 years	0-0.1 vs 0	0.913	0.726	1.147
10 - 19 years	0.1-0.4 vs 0	0.862	0.65	1.145
10 - 19 years	>0.4 vs 0	1.048	0.779	1.411
Manufacturers (Ref: 1 manufacturer)				
10+ manufacturers	0-0.1 vs 0	0.934	0.458	1.902

© 2026 Santhireswaran A et al. *JAMA Network Open*.

10+ manufacturers	0.1-0.4 vs 0	0.36	0.124	1.045
10+ manufacturers	>0.4 vs 0	0.199	0.078	0.508
2 - 4 manufacturers	0-0.1 vs 0	1.052	0.836	1.325
2 - 4 manufacturers	0.1-0.4 vs 0	0.766	0.57	1.03
2 - 4 manufacturers	>0.4 vs 0	0.545	0.394	0.754
5 - 9 manufacturers	0-0.1 vs 0	0.851	0.522	1.388
5 - 9 manufacturers	0.1-0.4 vs 0	0.251	0.118	0.534
5 - 9 manufacturers	>0.4 vs 0	0.119	0.043	0.333
Sales (Ref: 5 million+ sales)				
Less than 100k sales	0-0.1 vs 0	2.864	1.899	4.32
Less than 100k sales	0.1-0.4 vs 0	3.015	1.843	4.933
Less than 100k sales	>0.4 vs 0	5.594	3.53	8.865
100k - 499k sales	0-0.1 vs 0	2.034	1.374	3.011
100k - 499k sales	0.1-0.4 vs 0	1.603	0.998	2.576
100k - 499k sales	>0.4 vs 0	1.65	1.038	2.623
500k - 1.9 million sales	0-0.1 vs 0	2.199	1.518	3.185
500k - 1.9 million sales	0.1-0.4 vs 0	1.66	1.048	2.627
500k - 1.9 million sales	>0.4 vs 0	1.418	0.895	2.248
2 - 4.9 million sales	0-0.1 vs 0	1.339	0.895	2.003
2 - 4.9 million sales	0.1-0.4 vs 0	1.813	1.133	2.904
2 - 4.9 million sales	>0.4 vs 0	0.808	0.458	1.425
Number of Active Pharmaceutical Ingredients (APIs) (Ref: 1 API)				
2 APIs	0-0.1 vs 0	0.934	0.72	1.212
2 APIs	0.1-0.4 vs 0	0.99	0.728	1.348
2 APIs	>0.4 vs 0	1.972	1.471	2.642
3 APIs	0-0.1 vs 0	1.708	1.204	2.421
3 APIs	0.1-0.4 vs 0	1.761	1.144	2.712
3 APIs	>0.4 vs 0	1.58	0.942	2.651
4+ APIs	0-0.1 vs 0	0.947	0.563	1.592
4+ APIs	0.1-0.4 vs 0	0.744	0.379	1.459
4+ APIs	>0.4 vs 0	0.303	0.131	0.701
Number of Drug Identification Numbers (DINs) (Ref: 1 DIN)				
2 - 3 DINs	0-0.1 vs 0	0.774	0.612	0.979
2 - 3 DINs	0.1-0.4 vs 0	0.557	0.418	0.743
2 - 3 DINs	>0.4 vs 0	0.753	0.572	0.99
4 - 9 DINs	0-0.1 vs 0	0.702	0.534	0.923
4 - 9 DINs	0.1-0.4 vs 0	0.591	0.426	0.821

© 2026 Santhireswaran A et al. *JAMA Network Open*.

4 - 9 DINs	>0.4 vs 0	0.748	0.529	1.057
10 - 24 DINs	0-0.1 vs 0	0.673	0.445	1.017
10 - 24 DINs	0.1-0.4 vs 0	0.822	0.511	1.324
10 - 24 DINs	>0.4 vs 0	0.564	0.284	1.122
> 25 DINs	0-0.1 vs 0	1.14	0.63	2.06
> 25 DINs	0.1-0.4 vs 0	0.54	0.244	1.195
> 25 DINs	>0.4 vs 0	4.808	2.471	9.356
Ontario Drug Benefit (ODB) Coverage (Ref: No Coverage)				
1 - 49% DINs Covered	0-0.1 vs 0	0.365	0.252	0.531
1 - 49% DINs Covered	0.1-0.4 vs 0	0.536	0.333	0.861
1 - 49% DINs Covered	>0.4 vs 0	0.916	0.589	1.425
50 - 99% DINs Covered	0-0.1 vs 0	0.289	0.197	0.424
50 - 99% DINs Covered	0.1-0.4 vs 0	0.449	0.279	0.723
50 - 99% DINs Covered	>0.4 vs 0	0.655	0.429	0.999
100% DINs Covered	0-0.1 vs 0	0.398	0.293	0.539
100% DINs Covered	0.1-0.4 vs 0	0.823	0.585	1.158
100% DINs Covered	>0.4 vs 0	0.727	0.527	1.003
Schedule (Ref: Prescription)				
Ethical	0-0.1 vs 0	1.45	0.96	2.19
Ethical	0.1-0.4 vs 0	0.778	0.419	1.446
Ethical	>0.4 vs 0	0.896	0.503	1.597
Over-the-Counter	0-0.1 vs 0	3.069	2.191	4.298
Over-the-Counter	0.1-0.4 vs 0	5.91	3.801	9.19
Over-the-Counter	>0.4 vs 0	1.107	0.712	1.723
Schedule D	0-0.1 vs 0	0.977	0.657	1.453
Schedule D	0.1-0.4 vs 0	0.711	0.428	1.18
Schedule D	>0.4 vs 0	1.388	0.85	2.267
Schedule G	0-0.1 vs 0	0.667	0.159	2.797
Schedule G	0.1-0.4 vs 0	1.205	0.284	5.113
Schedule G	>0.4 vs 0	4.185	1.662	10.541
Targeted/Narcotic	0-0.1 vs 0	1.171	0.69	1.99
Targeted/Narcotic	0.1-0.4 vs 0	1.944	1.055	3.581
Targeted/Narcotic	>0.4 vs 0	2.581	1.545	4.312
Unit Price (Ref: Less than $0.50/unit)				
$0.50 - $0.99/unit	0-0.1 vs 0	1.357	0.992	1.857
$0.50 - $0.99/unit	0.1-0.4 vs 0	0.916	0.593	1.414
$0.50 - $0.99/unit	>0.4 vs 0	1.109	0.771	1.595
$1 - $4.99/unit	0-0.1 vs 0	0.702	0.475	1.039

© 2026 Santhireswaran A et al. *JAMA Network Open*.

$1 - $4.99/unit	0.1-0.4 vs 0	0.8	0.512	1.25
$1 - $4.99/unit	>0.4 vs 0	0.653	0.433	0.986
$5 - $19.99/unit	0-0.1 vs 0	1.069	0.704	1.625
$5 - $19.99/unit	0.1-0.4 vs 0	0.832	0.497	1.392
$5 - $19.99/unit	>0.4 vs 0	0.783	0.479	1.28
$20 - $99.99/unit	0-0.1 vs 0	1.88	1.167	3.028
$20 - $99.99/unit	0.1-0.4 vs 0	2.266	1.311	3.914
$20 - $99.99/unit	>0.4 vs 0	1.253	0.709	2.214
$100+/unit	0-0.1 vs 0	2.243	1.274	3.949
$100+/unit	0.1-0.4 vs 0	2.056	1.057	3.999
$100+/unit	>0.4 vs 0	3.436	1.851	6.378
Dominant Sector (Ref: Retail)				
Hospital	0-0.1 vs 0	0.934	0.662	1.319
Hospital	0.1-0.4 vs 0	1.642	1.087	2.48
Hospital	>0.4 vs 0	1.503	0.978	2.309
Both	0-0.1 vs 0	0.972	0.699	1.351
Both	0.1-0.4 vs 0	1.359	0.949	1.946
Both	>0.4 vs 0	1.932	1.42	2.627
World Health Organization (WHO) essential medicine	0-0.1 vs 0	0.673	0.534	0.849
World Health Organization (WHO) essential medicine	0.1-0.4 vs 0	0.956	0.727	1.256
World Health Organization (WHO) essential medicine	>0.4 vs 0	0.628	0.469	0.841
Therapeutic Equivilent	0-0.1 vs 0	0.951	0.73	1.239
Therapeutic Equivilent	0.1-0.4 vs 0	0.953	0.693	1.309
Therapeutic Equivilent	>0.4 vs 0	1.123	0.812	1.554
Tier 3	0-0.1 vs 0	1.069	0.687	1.664
Tier 3	0.1-0.4 vs 0	0.963	0.545	1.702
Tier 3	>0.4 vs 0	1.589	0.911	2.771
Previous Shortage	0-0.1 vs 0	1.012	0.823	1.245
Previous Shortage	0.1-0.4 vs 0	1.331	1.027	1.724
Previous Shortage	>0.4 vs 0	2.022	1.574	2.596
Brand	0-0.1 vs 0	0.576	0.397	0.835
Brand	0.1-0.4 vs 0	0.721	0.465	1.119
Brand	>0.4 vs 0	0.73	0.466	1.143

© 2026 Santhireswaran A et al. *JAMA Network Open*.

eFigure 4: Limited to Shortage-Events Excluding Discontinuations Sensitivity Analysis for Incident Shortage Outcome

Variable	OR (95% CI)
No Supply Chain Issue	0.404 (0.333, 0.489)
**Formulation**	
Parenteral	1 (Reference)
Inhaled	0.703 (0.448, 1.104)
Ophthalmic/Otic	1.253 (0.541, 2.901)
Oral	1.462 (1.107, 1.931)
Rectal	0.169 (0.038, 0.75)
Topical	1.463 (0.988, 2.166)
Transdermal	2.172 (1.01, 4.669)
Vaginal	0.648 (0.255, 1.643)
**Anatomical Therapeutic Class (ATC) Level–1**	
Alimentary tract & Metabolism	0.723 (0.532, 0.982)
Blood	1.495 (1.011, 2.212)
Cardiovascular	1.042 (0.759, 1.433)
Dermatologicals	0.701 (0.476, 1.032)
Genitourinary & Sex hormones	1.047 (0.712, 1.539)
Systemic hormonal	0.909 (0.553, 1.496)
Antiinfective	3.072 (2.335, 4.043)
Antineoplastic & Immunomodulating	0.737 (0.532, 1.022)
Musculoskeletal	1.406 (1.015, 1.948)
Nervous	1 (Reference)
Antiparasitic & Insecticides	1.234 (0.719, 2.116)
Respiratory	2.114 (1.619, 2.76)
Sensory	1.273 (0.582, 2.785)
Various	1.165 (0.678, 1.999)
**Unit Price**	
Less than $0.50/unit	1 (Reference)
$0.50 – $0.99/unit	1.216 (0.966, 1.531)
$1 – $4.99/unit	0.733 (0.567, 0.947)
$5 – $19.99/unit	0.979 (0.729, 1.314)
$20 – $99.99/unit	2.057 (1.482, 2.855)
$100+/unit	2.92 (1.981, 4.303)
**Age**	
Less than 5 years	1.017 (0.729, 1.421)
5 – 9 years	0.636 (0.471, 0.858)
10 – 19 years	0.909 (0.766, 1.078)
20+ years	1 (Reference)
**Manufacturers**	
1 manufacturer	1 (Reference)
2 – 4 manufacturers	0.701 (0.565, 0.87)
5 – 9 manufacturers	0.491 (0.316, 0.762)
10+ manufacturers	0.363 (0.184, 0.717)
**Sales**	
Less than 100k sales	4.449 (3.169, 6.245)
100k – 499k sales	2.231 (1.611, 3.09)
500k – 1.9 million sales	1.752 (1.284, 2.39)
2 – 4.9 million sales	1.224 (0.872, 1.719)
5 million+ sales	1 (Reference)
**Number of Active Pharmaceutical Ingredients (APIs)**	
1 API	1 (Reference)
2 APIs	1.188 (0.938, 1.504)
3 APIs	1.716 (1.207, 2.439)
4+ APIs	0.933 (0.541, 1.607)
**Number of Drug Identification Numbers (DINs)**	
1 DIN	1 (Reference)
2 – 3 DINs	0.803 (0.651, 0.991)
4 – 9 DINs	0.702 (0.544, 0.906)
10 – 24 DINs	0.576 (0.39, 0.852)
> 25 DINs	1.12 (0.661, 1.895)
**Ontario Drug Benefit (ODB) Coverage**	
0% DINs Covered	1 (Reference)
1 – 49% DINs Covered	0.666 (0.494, 0.898)
50 – 99% DINs Covered	0.505 (0.374, 0.681)
100% DINs Covered	0.602 (0.473, 0.766)
**Schedule**	
Ethical	0.996 (0.678, 1.462)
Over–the–Counter	2.726 (2.007, 3.703)
Schedule D	1.584 (1.112, 2.257)
Schedule G	1.977 (0.869, 4.5)
Targeted/Narcotic	1.279 (0.84, 1.949)
Prescription	1 (Reference)
**Dominant Sector**	
Hospital	1.649 (1.22, 2.228)
Retail	1 (Reference)
Both	1.88 (1.455, 2.43)
World Health Organization (WHO) essential medicine	0.961 (0.791, 1.168)
Therapeutic equivalent	1.009 (0.806, 1.264)
Tier–3	0.918 (0.626, 1.345)
Brand	0.578 (0.412, 0.809)
Previous supply chain issue	1.038 (0.851, 1.267)

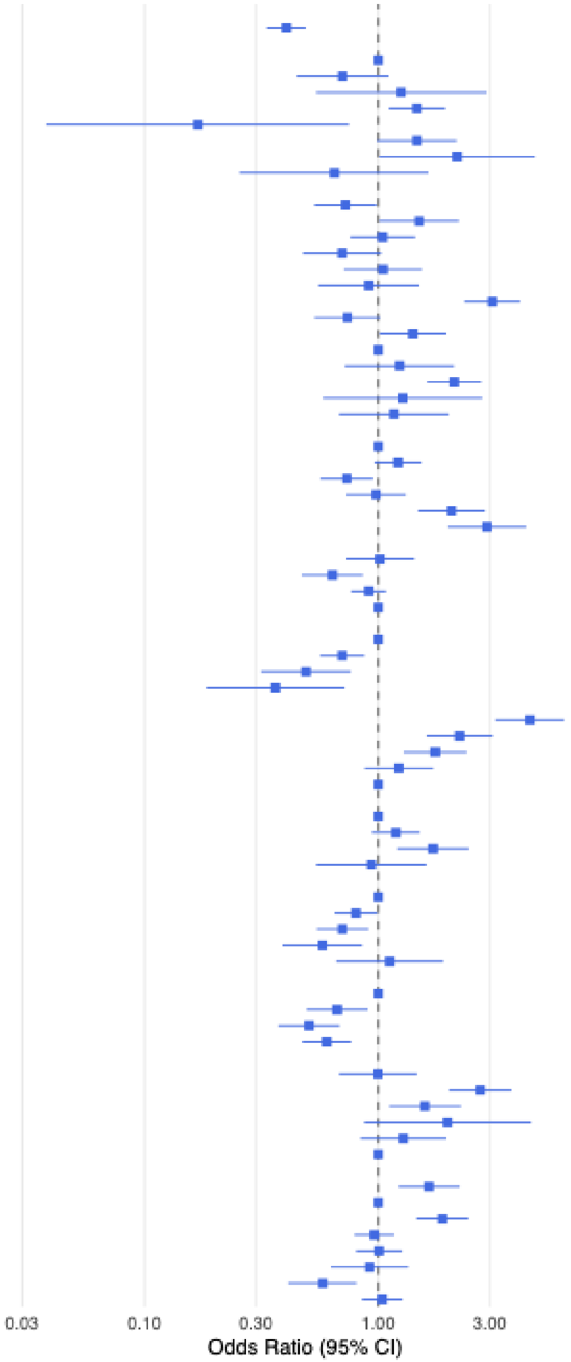

© 2026 Santhireswaran A et al. *JAMA Network Open*.

eFigure 5: Limited Number of Controls to 5 instead of 10 Sensitivity Analysis for Incident Shortage Outcome

Variable	OR (95% CI)
No Supply Chain Issue	0.344 (0.275, 0.431)
**Formulation**	
Parenteral	1 (Reference)
Inhaled	0.517 (0.274, 0.975)
Ophthalmic/Otic	1.792 (0.559, 5.742)
Oral	1.236 (0.841, 1.817)
Rectal	0.421 (0.086, 2.058)
Topical	1.491 (0.858, 2.593)
Transdermal	0.582 (0.12, 2.832)
Vaginal	0.383 (0.118, 1.245)
**Anatomical Therapeutic Class (ATC) Level–1**	
Alimentary tract & Metabolism	1.007 (0.644, 1.573)
Blood	1.61 (0.93, 2.789)
Cardiovascular	1.301 (0.827, 2.047)
Dermatologicals	0.874 (0.493, 1.55)
Genitourinary & Sex hormones	2.353 (1.426, 3.882)
Systemic hormonal	1.406 (0.76, 2.6)
Antiinfective	3.846 (2.579, 5.735)
Antineoplastic & Immunomodulating	0.936 (0.585, 1.498)
Musculoskeletal	1.704 (1.058, 2.744)
Nervous	1 (Reference)
Antiparasitic & Insecticides	1.553 (0.729, 3.31)
Respiratory	3.539 (2.364, 5.297)
Sensory	1.068 (0.357, 3.196)
Various	2.517 (1.371, 4.621)
**Unit Price**	
Less than $0.50/unit	1 (Reference)
$0.50 – $0.99/unit	1.295 (0.942, 1.779)
$1 – $4.99/unit	0.95 (0.678, 1.331)
$5 – $19.99/unit	0.967 (0.647, 1.445)
$20 – $99.99/unit	1.887 (1.207, 2.951)
$100+/unit	2.925 (1.754, 4.878)
**Age**	
Less than 5 years	0.606 (0.372, 0.985)
5 – 9 years	0.544 (0.356, 0.831)
10 – 19 years	0.724 (0.563, 0.931)
20+ years	1 (Reference)
**Manufacturers**	
1 manufacturer	1 (Reference)
2 – 4 manufacturers	0.873 (0.686, 1.11)
5 – 9 manufacturers	0.408 (0.24, 0.692)
10+ manufacturers	0.459 (0.215, 0.979)
**Sales**	
Less than 100k sales	3.634 (2.474, 5.339)
100k – 499k sales	1.803 (1.257, 2.586)
500k – 1.9 million sales	1.508 (1.069, 2.125)
2 – 4.9 million sales	1.093 (0.749, 1.595)
5 million+ sales	1 (Reference)
**Number of Active Pharmaceutical Ingredients (APIs)**	
1 API	1 (Reference)
2 APIs	1.325 (1.019, 1.722)
3 APIs	2.323 (1.562, 3.453)
4+ APIs	0.734 (0.403, 1.338)
**Number of Drug Identification Numbers (DINs)**	
1 DIN	1 (Reference)
2 – 3 DINs	0.658 (0.521, 0.833)
4 – 9 DINs	0.596 (0.449, 0.791)
10 – 24 DINs	0.549 (0.358, 0.842)
> 25 DINs	1.07 (0.595, 1.926)
**Ontario Drug Benefit (ODB) Coverage**	
0% DINs Covered	1 (Reference)
1 – 49% DINs Covered	0.537 (0.38, 0.758)
50 – 99% DINs Covered	0.42 (0.299, 0.59)
100% DINs Covered	0.533 (0.407, 0.699)
**Schedule**	
Ethical	1.139 (0.754, 1.723)
Over–the–Counter	2.288 (1.62, 3.231)
Schedule D	0.913 (0.615, 1.355)
Schedule G	1.228 (0.392, 3.85)
Targeted/Narcotic	1.515 (0.924, 2.483)
Prescription	1 (Reference)
**Dominant Sector**	
Hospital	1.151 (0.821, 1.614)
Retail	1 (Reference)
Both	1.208 (0.892, 1.634)
World Health Organization (WHO) essential medicine	0.969 (0.782, 1.201)
Therapeutic equivalent	1.074 (0.837, 1.38)
Tier–3	1.271 (0.851, 1.897)
Brand	0.811 (0.559, 1.176)
Previous supply chain issue	1.175 (0.955, 1.446)

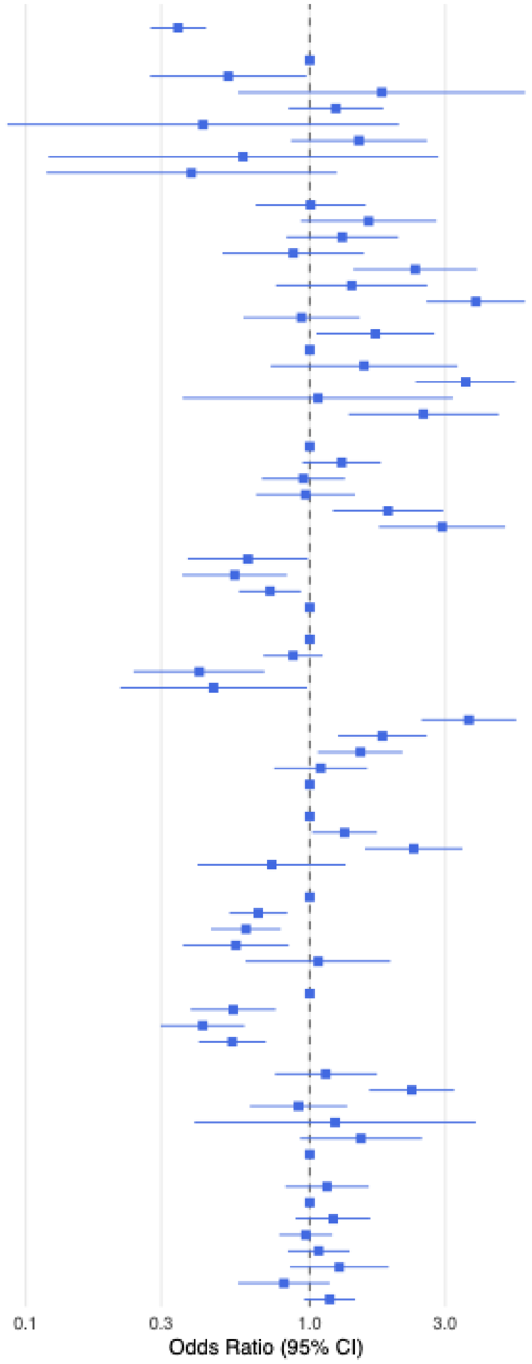

© 2026 Santhireswaran A et al. *JAMA Network Open*.

eFigure 6: Defining the Outcome within 9 months of the Supply Chain Event instead of 6 months Sensitivity Analysis for Incident Shortage Outcome

Variable	OR (95% CI)
No Supply Chain Issue	0.392 (0.325, 0.473)
**Formulation**	
Parenteral	1 (Reference)
Inhaled	0.703 (0.452, 1.092)
Ophthalmic/Otic	1.443 (0.639, 3.258)
Oral	1.543 (1.179, 2.02)
Rectal	0.233 (0.066, 0.819)
Topical	1.467 (1, 2.151)
Transdermal	1.999 (0.928, 4.31)
Vaginal	0.88 (0.381, 2.034)
**Anatomical Therapeutic Class (ATC) Level−1**	
Alimentary tract & Metabolism	0.783 (0.582, 1.053)
Blood	1.591 (1.085, 2.334)
Cardiovascular	1.217 (0.898, 1.649)
Dermatologicals	0.78 (0.534, 1.14)
Genitourinary & Sex hormones	1.06 (0.728, 1.543)
Systemic hormonal	0.98 (0.609, 1.577)
Antiinfective	3.34 (2.557, 4.364)
Antineoplastic & Immunomodulating	0.823 (0.6, 1.128)
Musculoskeletal	1.419 (1.028, 1.958)
Nervous	1 (Reference)
Antiparasitic & Insecticides	1.319 (0.78, 2.229)
Respiratory	2.268 (1.743, 2.952)
Sensory	1.305 (0.61, 2.795)
Various	1.162 (0.678, 1.99)
**Unit Price**	
Less than $0.50/unit	1 (Reference)
$0.50 – $0.99/unit	1.214 (0.97, 1.52)
$1 – $4.99/unit	0.823 (0.645, 1.051)
$5 – $19.99/unit	1.061 (0.8, 1.407)
$20 – $99.99/unit	2.241 (1.634, 3.074)
$100+/unit	3.23 (2.22, 4.698)
**Age**	
Less than 5 years	1.029 (0.743, 1.424)
5 – 9 years	0.696 (0.521, 0.929)
10 – 19 years	0.945 (0.802, 1.115)
20+ years	1 (Reference)
**Manufacturers**	
1 manufacturer	1 (Reference)
2 – 4 manufacturers	0.84 (0.712, 0.993)
5 – 9 manufacturers	0.492 (0.339, 0.714)
10+ manufacturers	0.626 (0.377, 1.039)
**Sales**	
Less than 100k sales	4.421 (3.374, 5.792)
100k – 499k sales	2.015 (1.554, 2.614)
500k – 1.9 million sales	1.911 (1.492, 2.449)
2 – 4.9 million sales	1.321 (1.012, 1.725)
5 million+ sales	1 (Reference)
**Number of Active Pharmaceutical Ingredients (APIs)**	
1 API	1 (Reference)
2 APIs	1.197 (1.002, 1.429)
3 APIs	1.981 (1.523, 2.577)
4+ APIs	0.661 (0.44, 0.992)
**Number of Drug Identification Numbers (DINs)**	
1 DIN	1 (Reference)
2 – 3 DINs	0.689 (0.586, 0.81)
4 – 9 DINs	0.657 (0.542, 0.796)
10 – 24 DINs	0.69 (0.514, 0.926)
> 25 DINs	1.136 (0.753, 1.714)
**Ontario Drug Benefit (ODB) Coverage**	
0% DINs Covered	1 (Reference)
1 – 49% DINs Covered	0.507 (0.395, 0.652)
50 – 99% DINs Covered	0.441 (0.346, 0.562)
100% DINs Covered	0.547 (0.451, 0.662)
**Schedule**	
Ethical	1.151 (0.839, 1.578)
Over−the−Counter	3.072 (2.422, 3.897)
Schedule D	1.067 (0.81, 1.407)
Schedule G	2.005 (1.048, 3.836)
Targeted/Narcotic	1.717 (1.228, 2.399)
Prescription	1 (Reference)
**Dominant Sector**	
Hospital	1.252 (0.986, 1.589)
Retail	1 (Reference)
Both	1.432 (1.165, 1.761)
World Health Organization (WHO) essential medicine	0.764 (0.652, 0.894)
Therapeutic equivalent	1.052 (0.879, 1.26)
Tier−3	1.305 (0.96, 1.775)
Brand	0.594 (0.46, 0.766)
Previous supply chain issue	1.325 (1.147, 1.53)

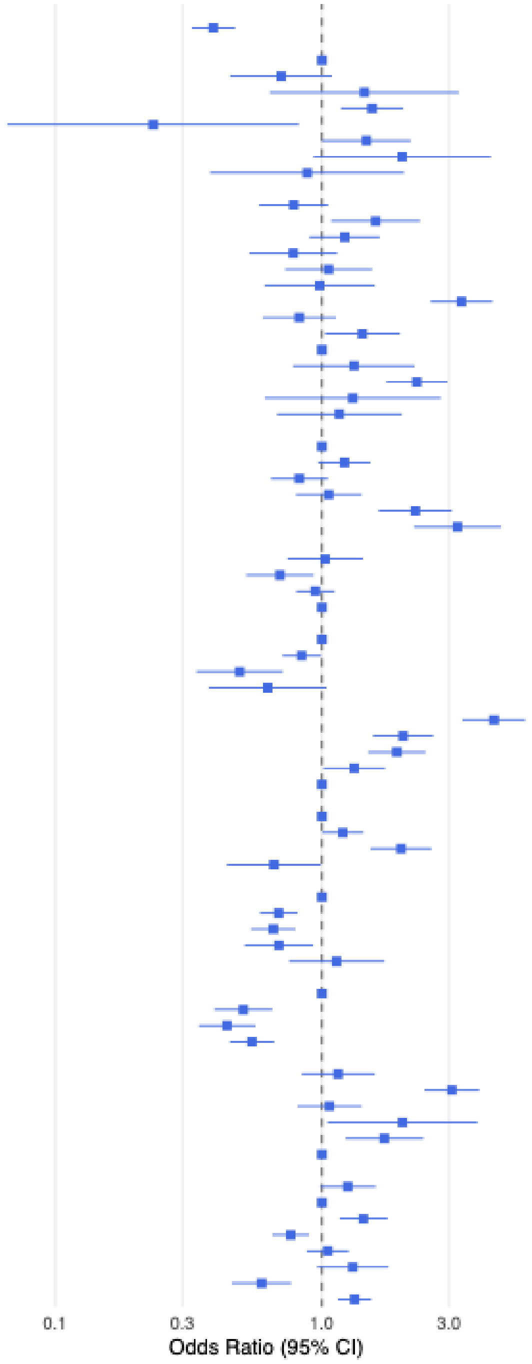

© 2026 Santhireswaran A et al. *JAMA Network Open*.

eFigure 7: Including the Post-COVID Pandemic Variable Sensitivity Analysis for Incident Shortage Outcome

Variable	OR (95% CI)
No Supply Chain Issue	0.404 (0.333, 0.489)
**Formulation**	
Parenteral	1 (Reference)
Inhaled	0.703 (0.448, 1.104)
Ophthalmic/Otic	1.253 (0.541, 2.901)
Oral	1.462 (1.107, 1.931)
Rectal	0.169 (0.038, 0.75)
Topical	1.463 (0.988, 2.166)
Transdermal	2.172 (1.01, 4.669)
Vaginal	0.648 (0.255, 1.643)
**Anatomical Therapeutic Class (ATC) Level–1**	
Alimentary tract & Metabolism	0.723 (0.532, 0.982)
Blood	1.495 (1.011, 2.212)
Cardiovascular	1.042 (0.759, 1.433)
Dermatologicals	0.701 (0.476, 1.032)
Genitourinary & Sex hormones	1.047 (0.712, 1.539)
Systemic hormonal	0.909 (0.553, 1.496)
Antiinfective	3.072 (2.335, 4.043)
Antineoplastic & Immunomodulating	0.737 (0.532, 1.022)
Musculoskeletal	1.406 (1.015, 1.948)
Nervous	1 (Reference)
Antiparasitic & Insecticides	1.234 (0.719, 2.116)
Respiratory	2.114 (1.619, 2.76)
Sensory	1.273 (0.582, 2.785)
Various	1.165 (0.678, 1.999)
**Unit Price**	
Less than $0.50/unit	1 (Reference)
$0.50 – $0.99/unit	1.216 (0.966, 1.531)
$1 – $4.99/unit	0.733 (0.567, 0.947)
$5 – $19.99/unit	0.979 (0.729, 1.314)
$20 – $99.99/unit	2.057 (1.482, 2.855)
$100+/unit	2.92 (1.981, 4.303)
**Age**	
Less than 5 years	1.017 (0.729, 1.421)
5 – 9 years	0.636 (0.471, 0.858)
10 – 19 years	0.909 (0.766, 1.078)
20+ years	1 (Reference)
**Manufacturers**	
1 manufacturer	1 (Reference)
2 – 4 manufacturers	0.849 (0.716, 1.008)
5 – 9 manufacturers	0.455 (0.307, 0.674)
10+ manufacturers	0.561 (0.33, 0.955)
**Sales**	
Less than 100k sales	4.016 (3.036, 5.313)
100k – 499k sales	1.884 (1.439, 2.467)
500k – 1.9 million sales	1.875 (1.45, 2.426)
2 – 4.9 million sales	1.305 (0.988, 1.724)
5 million+ sales	1 (Reference)
**Number of Active Pharmaceutical Ingredients (APIs)**	
1 API	1 (Reference)
2 APIs	1.206 (1.005, 1.448)
3 APIs	1.858 (1.417, 2.436)
4+ APIs	0.679 (0.449, 1.025)
**Number of Drug Identification Numbers (DINs)**	
1 DIN	1 (Reference)
2 – 3 DINs	0.695 (0.588, 0.822)
4 – 9 DINs	0.682 (0.56, 0.831)
10 – 24 DINs	0.7 (0.516, 0.949)
> 25 DINs	1.268 (0.831, 1.935)
**Ontario Drug Benefit (ODB) Coverage**	
0% DINs Covered	1 (Reference)
1 – 49% DINs Covered	0.503 (0.388, 0.651)
50 – 99% DINs Covered	0.405 (0.314, 0.523)
100% DINs Covered	0.557 (0.456, 0.68)
**Schedule**	
Ethical	1.124 (0.811, 1.557)
Over–the–Counter	3.054 (2.387, 3.906)
Schedule D	1.015 (0.763, 1.351)
Schedule G	1.605 (0.786, 3.275)
Targeted/Narcotic	1.784 (1.271, 2.503)
Prescription	1 (Reference)
**Dominant Sector**	
Hospital	1.324 (1.035, 1.694)
Retail	1 (Reference)
Both	1.468 (1.187, 1.816)
World Health Organization (WHO) essential medicine	0.744 (0.631, 0.877)
Therapeutic equivalent	1.016 (0.843, 1.224)
Tier–3	1.167 (0.845, 1.611)
Brand	0.655 (0.504, 0.853)
Previous supply chain issue	1.41 (1.212, 1.639)
Post–COVID	0.918 (0.787, 1.07)

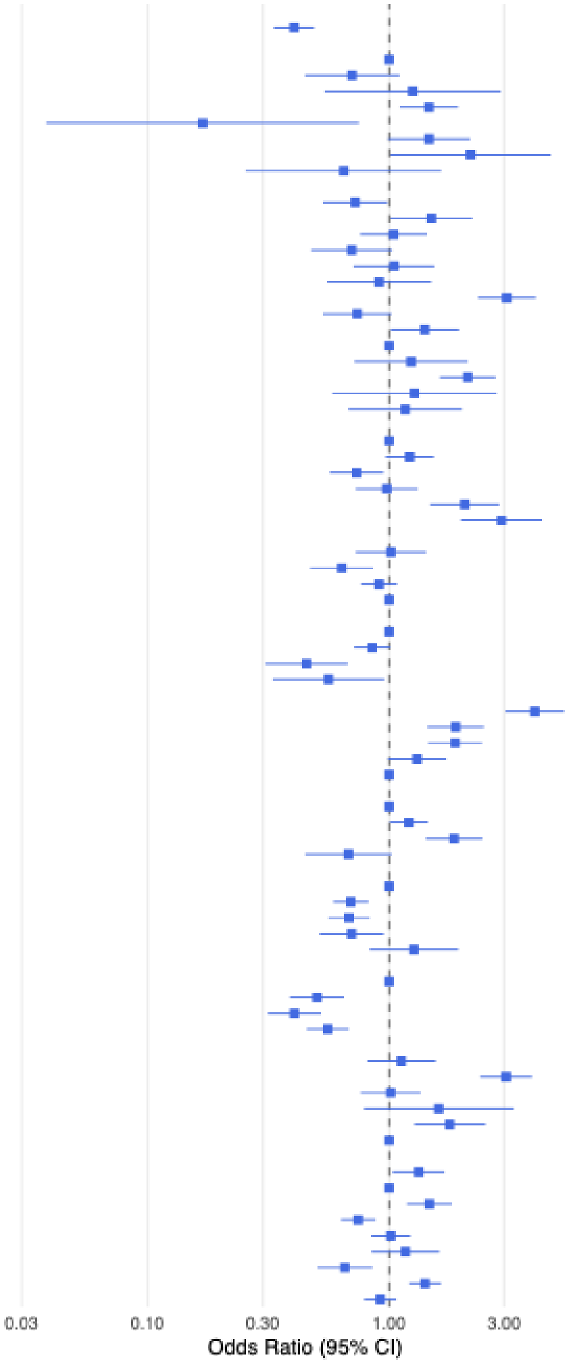

© 2026 Santhireswaran A et al. *JAMA Network Open*.

eFigure 8: Multivariate Regression Sensitivity Analysis for Shortage Intensity Outcome

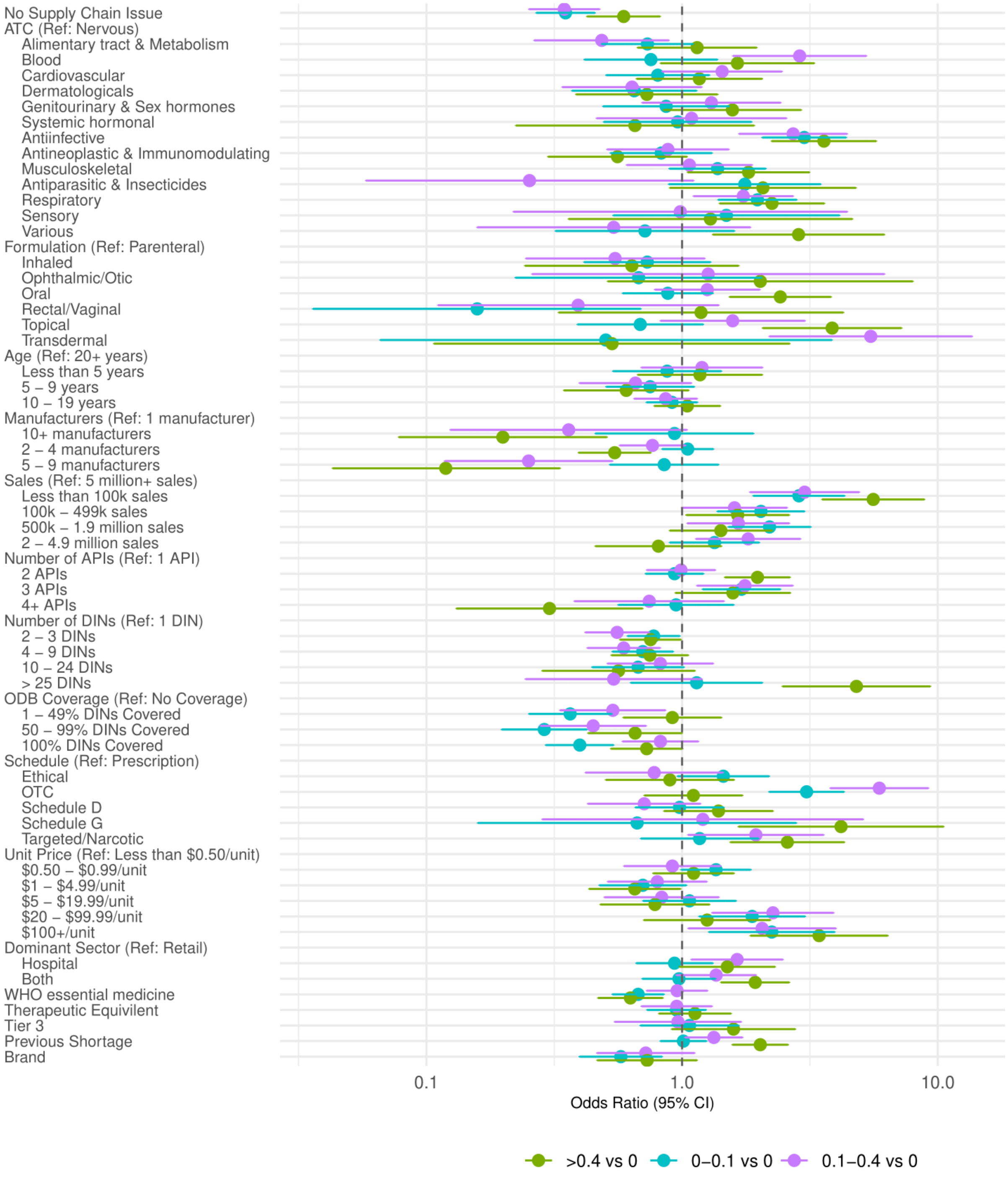

Odds ratios shown in Table S8 given space constraints.

eReferences

1. Ontario Ministry of Finance. Ontario Demographic Quarterly: highlights of second quarter. https://www.ontario.ca/page/ontario-demographic-quarterly-highlights-second-quarter
2. Ontario Ministry of Health. Ontario Drug Benefit Formulary Search. https://www.formulary.health.gov.on.ca/formulary/
3. Callaway Kim K, Rothenberger SD, Tadrous M, et al. Drug Shortages Prior to and During the COVID-19 Pandemic. *JAMA Netw Open*. Apr 1 2024;7(4):e244246. doi:10.1001/jamanetworkopen.2024.4246
4. Tadrous M, Callaway Kim K, Hernandez I, et al. Differences in Drug Shortages in the US and Canada. *Jama*. Dec 10 2024;332(22):1912-1922. doi:10.1001/jama.2024.17688
5. Gross AE, Johannes RS, Gupta V, Tabak YP, Srinivasan A, Bleasdale SC. The Effect of a Piperacillin/Tazobactam Shortage on Antimicrobial Prescribing and Clostridium difficile Risk in 88 US Medical Centers. *Clin Infect Dis*. Aug 15 2017;65(4):613-618. doi:10.1093/cid/cix379
6. Hu S, Santhireswaran A, Chu C, et al. The association between drug shortages and prices across 74 countries: uncovering global access inequities. *BMJ Glob Health*. Nov 23 2025;10(11)doi:10.1136/bmjgh-2025-018960

© 2026 Santhireswaran A et al. *JAMA Network Open*.
