# Supplementary material for: Supply Chain Events and Risk of Drug Shortage in Canada
Source: JAMA Netw Open. 2026 Jun 4;9(6):e2616632. doi: 10.1001/jamanetworkopen.2026.16632 (PMC13237616; doi:10.1001/jamanetworkopen.2026.16632)
Supplement: Supplement 2. — Data Sharing Statement [file jamanetwopen-e2616632-s002.pdf]

Data Sharing Statement

Santhireswaran. Supply Chain Events and Risk of Drug Shortage in Canada. *JAMA Netw Open*. Published June 04, 2026. doi:10.1001/jamanetworkopen.2026.16632

Data
Data available: No

Additional Information
Explanation for why data not available: The drug purchasing data used in this study were obtained from IQVIA under license and cannot be shared by the authors. These data may be obtained directly from IQVIA upon request and purchase. The ICES Drug List was accessed through ICES due to institutional affiliations and cannot be shared. Other data sources used in this study (Health Canada Drug Product Database, Drug Shortages Canada, WHO Essential Medicines List, and the Health Canada Tier-3 Drug List) are publicly available.
